# The 5′-Isobutyryl Ester Prodrug of 4′-Thiouridine Inhibits SARS-CoV‑2 Replication in Culture and in a Syrian Hamster Infection Model

**DOI:** 10.1021/acsomega.6c00102

**Published:** 2026-07-10

**Authors:** Zhe Chen, Franck Amblard, Mahesh Kasthuri, Zahira Tber, Julia C. LeCher, Sijia Tao, Ramyani De, Ingrid Marko, Leda Bassit, Keivan Zandi, Tamara McBrayer, Selwyn H. Hurwitz, Birgit Weynand, Rana Abdelnabi, Johan Neyts, Siddhartha Bhatt, Britton Boras, Rhonda D. Cardin, Yuao Zhu, Mili Kapoor, Heather Eng, Amit S. Kalgutkar, Siennah R. Greenfield, Rhishikesh Thakare, Amanda King-Ahmad, Nandini C. Patel, Kenneth A. Johnson, David Hepworth, Andrew Fensome, Raymond F. Schinazi

**Affiliations:** † Center for ViroScience and Cure, Laboratory of Biochemical Pharmacology, Department of Pediatrics, Emory University School of Medicine and Children’s Healthcare of Atlanta, Atlanta, Georgia 30322, United States; ‡ Department of Microbiology, Immunology and Transplantation, Rega Institute, Virology, Antiviral Drug & Vaccine Research Group, 26657 KU Leuven, Leuven 3000, Belgium; § Department of Microbiology, Immunology and Transplantation, KU Leuven, VirusBank Platform, Leuven 3000, Belgium; ∥ Department of Molecular Biosciences, 12330 University of Texas at Austin, Austin, Texas 78712, United States; ⊥ Department of Imaging and Pathology, Translational Cell and Tissue Research, Division of Translational Cell and Tissue Research, KU Leuven, Leuven 3000, Belgium; # Research and Development, Pfizer, Groton, Connecticut 06340, United States; ¶ Research and Development, Pfizer, La Jolla, California 92121, United States; ∇ Research and Development, Pfizer, Pearl River, New York 10965, United States; ○ Research and Development, Pfizer, Cambridge, Massachusetts 02139, United States

## Abstract

Although the SARS-CoV-2 pandemic is now largely controlled through widespread vaccination and available therapeutics, emerging variants could undermine their overall effectiveness. 4′-Thiouridine (**TU**) and its 5′-isobutyryl prodrug (*iBu* **TU**) were identified as potent inhibitors of SARS-CoV-2 replication across various cell models, including advanced primary human respiratory models. Both compounds exhibited no significant toxicity across a range of cell culture systems, with no signs of mitochondrial damage or elevated lactic acid production. Furthermore, **TU** and *iBu* **TU** tested negative for mutagenicity, aneugenic and clastogenic effects, alleviating concerns about genotoxicity. They also showed no interaction with a broad spectrum of ion channels, GPCRs, and enzymes, further confirming their favorable safety profile. **TU** is metabolized intracellularly to its active triphosphate form (**TU**-TP), which effectively disrupts viral RNA replication by acting as a delayed chain terminator. *iBu* **TU** is rapidly converted to **TU** in plasma and displays superior oral bioavailability compared to **TU** (*F* = 90% vs 42% in monkeys). In a Syrian hamster model infected with SARS-CoV-2, oral *iBu* **TU** significantly reduced viral loads (1.7 to 4.5 log_10_) and improved lung health without adverse effects. These findings highlight *iBu* **TU** as a promising oral antiviral candidate for treating SARS-CoV-2 infection.

## Graphical abstract

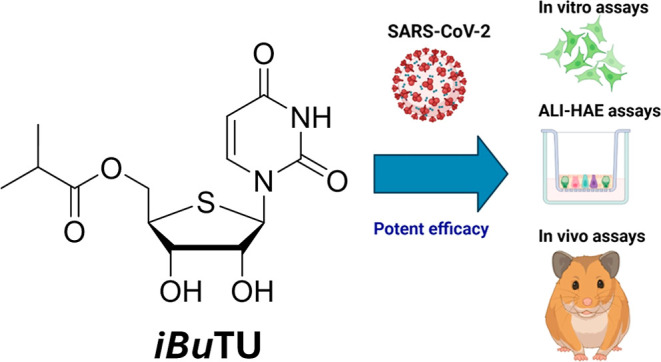

## Introduction

As a member of the coronavirus family, severe acute respiratory syndrome coronavirus 2 (SARS-CoV-2) is an enveloped, positive-sense, single-stranded RNA virus, first identified in 2019 in the city of Wuhan, China. In early 2020, the virus spread rapidly in China and other countries, leading to a global pandemic with unprecedented socio-economic repercussions and, more importantly, major public health consequences. From 2020 to 2025, the virus infected more than 770 million people and led to over 7 million deaths.^1^ In response to the pandemic, vaccines and therapeutics were developed; compounds approved by the U.S. Food and Drug Administration (FDA) include the SARS-CoV-2 main protease (M^pro^) inhibitor nirmatrelvir administered in combination with ritonavir,^2^ the RNA-dependent RNA polymerase (RdRp) inhibitors remdesivir and molnupiravir. New RdRp inhibitors, such as obeldesivir^3^ (now discontinued for SARS-CoV-2 infection), mindeudesivir/VV116,^4^ and CNC/CNiBuC^5^ along with next generation M^pro^ inhibitors such as ibuzatrelvir^6^ (Figure 1), are currently being evaluated in the US and/or China. Herein, we report the discovery and evaluation of 4′-thiouridine (**TU** and its 5′-isobutyryl prodrug (*iBu* **TU**)) as promising nucleoside analogs, inhibitors of SARS-CoV-2 RNA-dependent RNA polymerase (RdRp) with potent activity both in vitro and in vivo.

**Figure 1:**
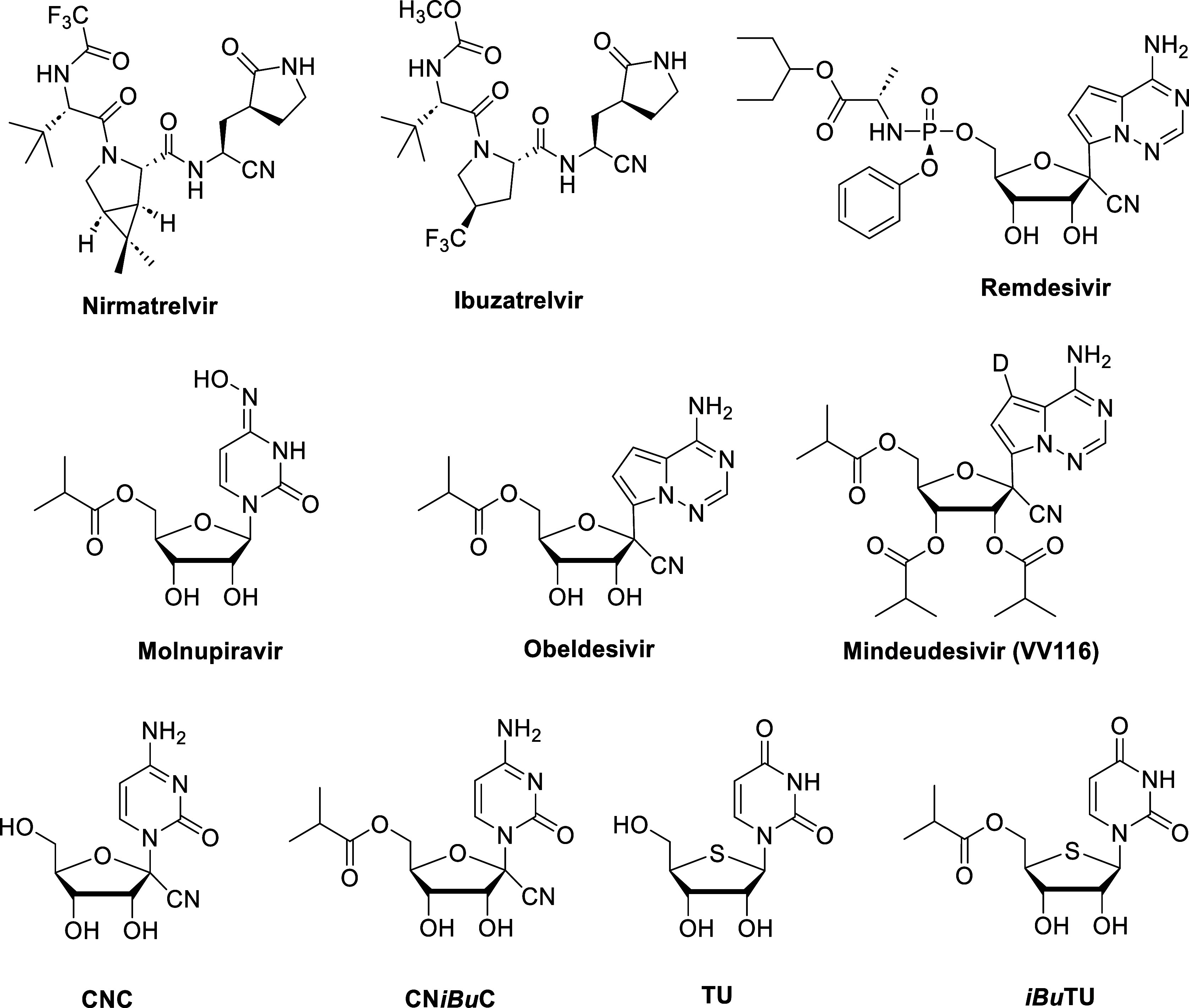
Chemical structures of 4′-thiouridine (**TU**), its 5′-isobutyryl prodrug (*iBu* **TU**), and representative clinically approved or investigational anti-SARS-CoV-2 agents.

While this manuscript was being prepared, a group in South Korea led by Kim et al. published in June, 2025, a similar paper on the discovery of **TU**’s activity against SARS-CoV-2 in culture.^7^ It is noteworthy that this compound was first invented and disclosed in our U.S. Patent application (No. US20240216413A1), filed on April 11, 2022, which describes the anti-SARS-CoV-2 activity of **TU**. This patent predates their publication by over three years and was not cited in their manuscript, likely due to an oversight.

## Results and Discussion

### Evaluation of 4′-Thiouridine against SARS-CoV-2 (Washington Strain) in Cell Culture

4′-Thiouridine (**TU**) and its 5′-isobutyryl prodrug (*iBu* **TU**) were initially evaluated against the SARS-CoV-2 root-lineage Washington strain in various cell systems, including African Green monkey kidney cells (Vero), human lung adenocarcinoma cells (Calu-3), and human intestinal epithelial cells (Caco-2). Caco-2 cells were selected because it has been reported that SARS-CoV-2 infection can, in some instances, result in gastrointestinal symptoms such as diarrhea, vomiting, or abdominal pain. In these three cell systems, **TU** displayed EC_50_s in the low to submicromolar range similar to that of NHC, the parent compound of molnupiravir (Table 1)

**Table 1:** Anti-SARS-CoV-2 (Washington Strain) of 4′-Thiouridine in Vero, Calu-3 and Caco-2 Cells

	anti-SARS-CoV-2 activity^a^ (Vero)^b^ (μM)		anti-SARS-CoV-2 activity^a^ (Calu-3)^b^ (μM)		anti-SARS-CoV-2 activity^a^ (Caco-2)^b^ (μM)	
compound	EC_50_ ^c^	EC_90_ ^c^	EC_50_ ^c^	EC_90_ ^c^	EC_50_ ^c^	EC_90_ ^c^
**TU**	1.7 ± 0.5	2.4 ± 0.2	3.9 ± 0.2	4.9 ± 0.02	3.3 ± 1.3	6.4 ± 2.8
*iBu* **TU**	9.5 ± 1.1	16.4 ± 2.7	16.8 ± 5.2	43.6 ± 1.1	14.1 ± 4.2	93.8 ± 0.1
NHC	0.5 ± 0.09	1.4 ± 0.3	1.3 ± 0.5	2.9 ± 0.2	1.0 ± 0.7	5.1 ± 2.1
remdesivir	3.2 ± 0.8	4.7 ± 0.2	0.2 ± 0.004	0.6 ± 0.03	0.02 ± 0.003	0.1 ± 0.07

a Detection and quantification of SARS-CoV-2 genomic RNA in infected cell supernatant via qRT-PCR.

b Vero = African green monkey kidney cells. Calu-3 = human lung adenocarcinoma cells. Caco-2 = human intestinal colorectal adenocarcinoma cells.

c EC = median 50% (EC_50_) or 90% (EC_90_) effective antiviral concentration. ND = not determined. All values are shown as mean ± standard deviation. For cytotoxicity data see Table 4.

### In Vitro Evaluation of 4′-Thiouridine against SARS-CoV-2 Variants of Concern

Multiple SARS-CoV-2 variants of concern (VOCs) surfaced throughout the COVID-19 pandemic, exhibiting increased infectivity, transmissibility, and immune evasion. Notably, the Omicron variant emerged as the dominant strain worldwide, distinguished by a multitude of mutations in the spike protein that improve its binding affinity to the ACE-2 receptor.^8–13^ Thus, 4′-thiouridine was evaluated against several VOCs, including Alpha, Beta, Gamma, Delta, and Omicron, in both Vero (Alpha, Beta, Gamma) and Calu-3 cells (all 5). As shown in Table 2, **TU** appeared to be up to 3.5 times more potent against some of these variants than it was against the original Washington strain, with EC_50_s between 0.4 and 1.2 μM.

**Table 2:** Evaluation of **TU** and *iBu* **TU** against SARS-CoV-2 Variants of Concern in Vero and Calu-3 Cells^a^

	antiviral activity against SARS-CoV-2 Variants in Vero (μM)				antiviral activity against SARS-CoV-2 variants in Calu-3 (μM)					
	TU		remdesivir		TU		*iBu*TU		remdesivir	
SARS-CoV-2 strain	EC_50_	EC_90_	EC_50_	EC_90_	EC_50_	EC_90_	EC_50_	EC_90_	EC_50_	EC_90_
alpha	0.5 ± 0.3	2.0 ± 1.9	0.4 ± 0.5	0.9 ± 0.5	0.5 ± 0.3	2.8 ± 1.5	0.8 ± 0.04	6.8 ± 0.4	0.2 ± 0.1	0.7 ± 0.6
beta	0.7 ± 0.5	0.9 ± 0.5	0.7 ± 0.6	0.8 ± 0.7	1.0 ± 0.2	1.2 ± 0.2	0.06 ± 0.01	0.2 ± 0.05	0.2 ± 0.1	0.3 ± 0.1
gamma	0.4 ± 0.3	1.7 ± 1.4	0.3 ± 0.7	0.5 ± 0.6	0.4 ± 0.4	1.2 ± 1.5	2.6 ± 0.06	3.5 ± 0.4	0.9 ± 1.3	1.2 ± 1.6
delta	#	#	#	#	1.2 ± 0.9	3.2 ± 1.6	2.5 ± 0.1	3.5 ± 1.0	0.4 ± 0.2	1.5 ± 0.8
omicron	#	#	#	#	0.9 ± 0.2	2.5 ± 0.5	1.2 ± 0.4	10.4 ± 2.6	0.3 ± 0.3	0.5 ± 0.2

a #Values not determined due to limited replication of delta and omicron in Vero cells. All values are shown as mean ± standard deviation.

### Evaluation of TU in Primary Human Respiratory 3D Culture Models

Cellular 3D models have become increasingly valuable in the identification of potential new drugs, as they offer a more accurate representation of in vivo conditions. Our group recently reported the use of two novel 3D systems permissive to SARS-CoV-2 infection to evaluate CNC, a potent nucleoside analog inhibitor of SARS-CoV-2 RdRp.^6^ Thus, **TU** was evaluated in human bronchial/tracheal epithelial cultures grown at air–liquid interface (HBTEC-ALI) and in human bronchial/tracheal epithelial cultures cultivated as apical-out organoids (HBTEC-hAORBs),^14^ two systems in which it displayed submicromolar EC_50_s similar to that of control compound Remdesivir (Table 3).

### Cytotoxicity Profile

The cytotoxic effect of **TU** was evaluated in various cell lines, including human peripheral blood mononuclear (PBM) cells, human lymphoblasts (CEM), Vero, human hepatomas (Huh-7), Calu-3, and Caco-2 cells. In none of these cell systems did **TU** display any relevant toxicity, unlike controls NHC and remdesivir (Table 4).

Mitochondrial toxicity can pose a considerable risk in the development of new antiviral drugs as it may result in severe health complications in patients, including pancreatitis, hepatic steatosis, and lactic acidosis.^15^ However, neither **TU** nor *iBu* **TU** displayed mitochondrial toxicity at concentrations up to 50 μM in human hepatoma (HepG2) cells in a 14 day assay and none of them induced an increase in lactic acid production (% of lactic acid/% of nuclear DNA).^16^

In addition, chromosomal damage was assessed in the A375 human melanoma cell line, in a high content imaging format.^17^ Both *iBu* **TU** and **TU** were negative for markers of aneugenicity or clastogenicity. In a non-GLP Ames assay, *iBu* **TU** and **TU** were negative in TA1535, TA1537 and TA98 strains with and without rat S9 metabolic activation derisking potential mutagenicity and genotoxicity issues.

Polypharmacology was also assessed for both *iBu* **TU** and **TU** against a panel of ion channels, GPCRs, and enzymes (Supporting Information Table S2). In automated patch clamp assays for the hERG (HEK-293) and NaV1.5 (CHO) ion channels, neither compound had activity below 100 μM. In a radiolabeled dofetilide binding assay, both compounds had IC_50_ > 90 μM. *iBu* **TU** and **TU** had IC_50_s above 30 μM against a panel of GPCRs overexpressed in either CHO or HEK-293 cells, in both agonist and antagonist modes. Both compounds were inactive in assays for phosphodiesterase isoforms PDE3B or PDE4D2 and acetylcholine esterase, with IC_50_ > 30 μM. Kinase cross reactivity of the compound was assessed (Invitrogen/Thermo-Fischer) at 10 μM, with ATP at either the *K*_m_ for the target kinase (36 targets), or at a pseudophysiological concentration of 1 mM (37 targets) (Supporting Information Table S3). Under conditions with the ATP concentration at the *K*_m_ value for each kinase, the only significant activity was for **TU** against MAP4K4 (43.3% inhibition). Under the 1 mM ATP test concentration, neither compound displayed significant activity against any of the target kinases.

**Table 3:** Anti-SARS-CoV-2 Activity of **TU** in Primary Human Respiratory Culture Systems

	anti-SARS-CoV-2 activity (μM) in primary 3D human respiratory models			
	HBTECs ALI^a^		HBTECs hAORB^b^	
compound	EC_50_	EC_90_	EC_50_	EC_90_
**TU**	0.6 ± 1.0	1.0 ± 0.9	0.9 ± 0.4	2.1 ± 0.5
remdesivir	0.3 ± 0.3	0.8 ± 0.7	0.4 ± 0.08	1.1 ± 0.7

a HBTEC-ALI = human bronchial/tracheal epithelial cultures grown at ALI.

b HBTEC-hAORBs = human bronchial/tracheal epithelial cultures cultivated as apical-out organoids with reversed biopolarity. All values are shown as mean ± standard deviation.

#### Cellular Pharmacology

The cellular metabolism of **TU** was evaluated in different cell systems^18^ and as expected for a nucleoside analog, **TU** was intracellularly metabolized to its corresponding 5′-triphosphate form (**TU**-TP) in HBTEC-ALI, Vero, Calu-3 and Caco-2 cells (Figure 2).

**Figure 2:**
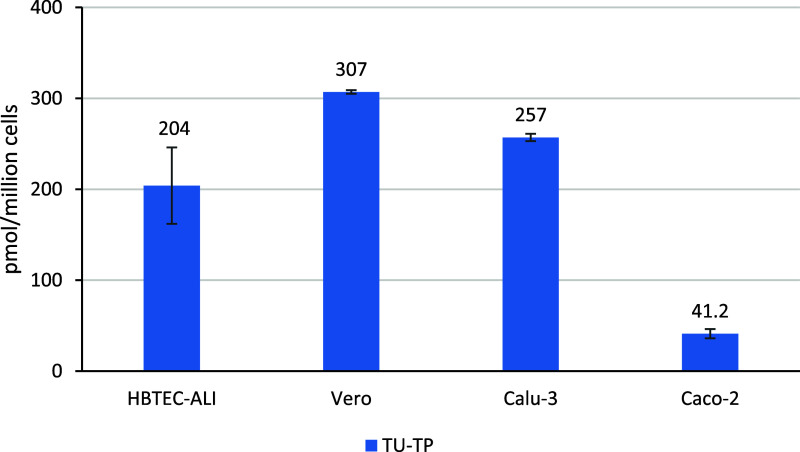
Intracellular accumulation of **TU**-TP after 4 h incubation of **TU** at 10 μM in HBTEC-ALI, Vero, Calu-3 and Caco-2 cells.

**Table 4:** Cytotoxicity of TU and *iBu*TU Relative to Other anti-SARS-CoV-2 Agents in PBM, CEM, Vero, Huh-7, Caco-2, and Calu-3 Cells

	cytotoxicity CC_50_ (μM)					
compound	PBM	CEM	Vero	Huh-7	Caco-2	Calu-3
**TU**	>100	45.9 ± 18.3	>100	>100	>100	>100
*iBu* **TU**	>100	>100	>100	>100	>100	>100
NHC	49.2 ± 26.4	2.4 ± 0.8	24.6 ± 20.4	40.6 ± 27.9	>100	>100
remdesivir	3.6 ± 1.8	7.7 ± 4.2	>100	5.6 ± 6.6	>100	>100

### Biochemistry

#### Kinetic Analysis Reveals TU-TP is a Delayed Chain Terminator of SARS-CoV-2 Replication

To determine the kinetic basis for inhibition of SARS-CoV-2 replication by **TU**-TP, presteady-state incorporation experiments with purified RdRp (NSP12/7/8 complex) were performed (Figure 3). Single turnover time courses across a range of nucleotide concentrations were globally fit in KinTek explorer software^19,20^ to the model in Figure 3c. This analysis yielded the *K*_d,apparent_ (1/*K*_1_) for **TU**-TP binding, estimates of the forward and reverse chemistry rate constants (*k*_2_ and *k*_–2_) and rate of pyrophosphate release (*k*_3_). Confidence contour analysis was used as a rigorous test of the model and to estimate the upper and lower limits for each kinetic parameter (Supporting Information Figure S4).

**Figure 3:**
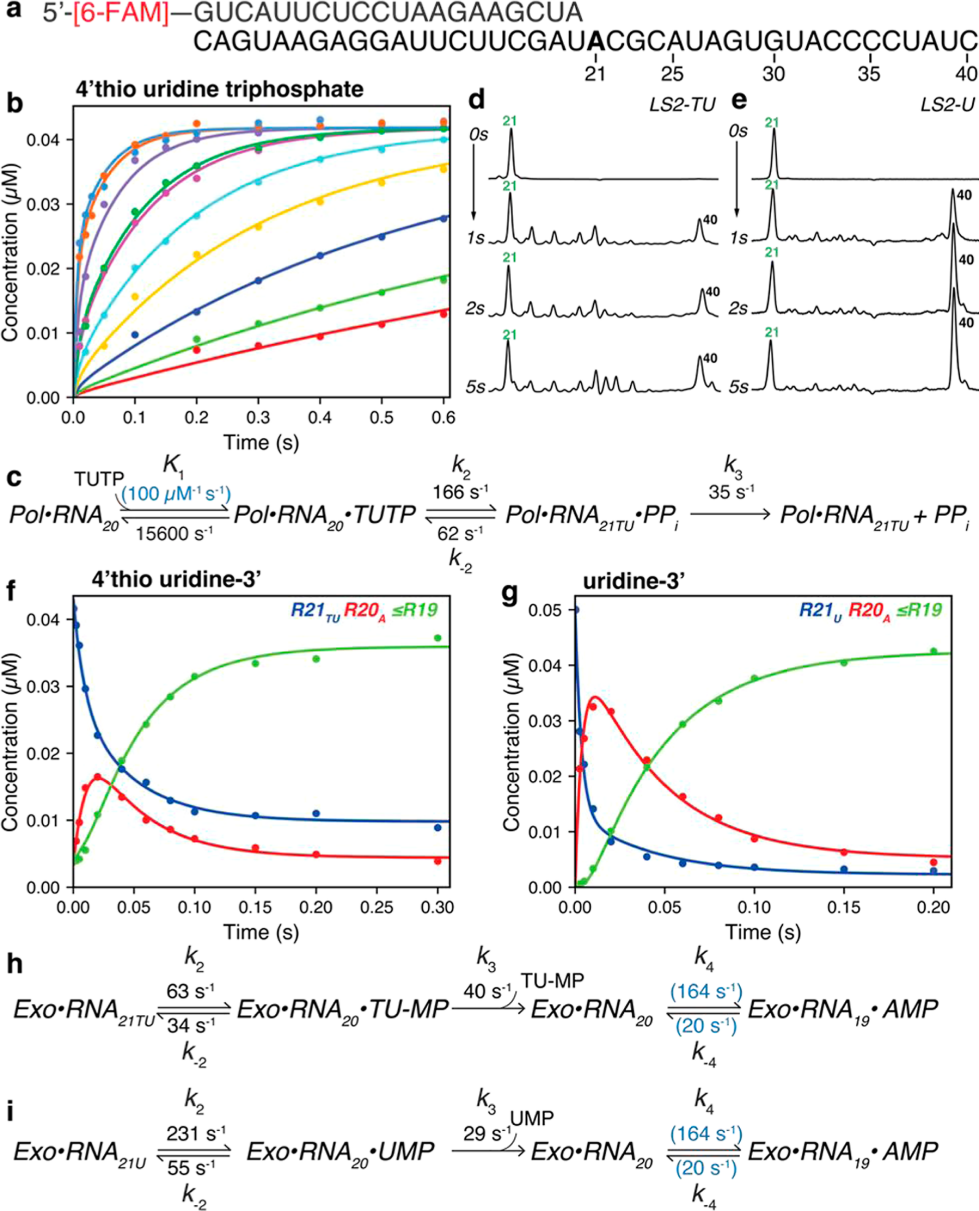
Mechanisms of 4′-thiouridine triphosphate incorporation and excision by SARS-CoV-2 polymerase and exonuclease complexes. (a) RNA substrate used in RdRp assays using a 5′-[6FAM]-labeled primer for quantification. The templating adenosine at position 21 (bold) specifies the nucleotide incorporation. (b) Presteady-state kinetics of **TU**-TP incorporation by the RdRp across a range of nucleotide concentrations. (c) Global fitting of the single-turnover data to the kinetic model for **TU**-TP incorporation using KinTek Explorer.^19,20^ Fixed parameters (blue) were previously reported;^5,21^ remaining rate constants were determined with fitting by simulation (black). The rate constant *k*_2_ was fixed at its best-fit value, as described in the text. (d,e) Capillary electrophoresis of RdRp extension of dsRNA with 4′-thiouridine (d) or uridine (e) at the 3′-end of the primer strand. Reactions were initiated with 100 μM NTPs in Mg^2+^ containing buffer and quenched with EDTA at the indicated time points. The electropherograms were scaled to the initial 21 nt peak height to enable comparison. (f,g) Presteady-state kinetics of **TU**-MP (f) or UMP (g) excision by NSP10/14 from the 3′-terminus of the RNA primer. Time courses show loss of R21 (blue), appearance and decay of R20 (red) intermediate, and accumulation of shorter products ≤19 nucleotides (green). (h,i) Global fitting of the excision data to kinetic models including reversible chemistry and slow NMP release steps. Rate constants (black) were fit using the fixed parameters (blue) from previously reported experiments derived from earlier experiments starting with R20.^5^ The substrate amplitude constrained the equilibrium constant for Exo·R21 complex formation (Supporting Information Figure S5).

This analysis showed that *k*_2_ and *k*_–2_ are correlated, with their equilibrium constant (*K*_2_) well-defined; each rate constant a had clear lower limit and best-fit value, but no upper limit. Fixing *k*_2_ at its best-fit value allowed definition of *k*_–2_, enabling more accurate calculations of the steady-state parameters shown in Table 5. Notably, NSP12 exhibits a lower *K*_m_ for **TU**-TP than UTP (Table 5), reflecting slow pyrophosphate release and reversible chemistry that allows nucleotide binding to approach equilibrium.^5^ This enhancement is modest (∼2-fold) because *k*_2_ > *k*_–2_, causing the incorporation reaction to be favorable, unlike the behavior observed for 1′-cyano-cytidine-triphosphate.^5^ The specificity constant (*k*_cat_/*K*_m_) for **TU**-UTP (0.38 μM^–1^ s^–1^) is lower than that of the natural substrate UTP (2.3 μM^–1^ s^–1^),^21^ yet remains 3 orders of magnitude higher than sofosbuvir for the HCV polymerase.^22^ At a hypothetical **TU**-TP concentration of 10 μM, the discrimination index and physiological UTP concentration (570 ± 460 μM)^23^ predict analog incorporation once every 70–600 adenosines in the template (using eq S5), a frequency well within the range for effective antiviral activity.

**Table 5:** Kinetic Parameters for Nucleotide Incorporation by SARS-CoV-2 NSP12/7/8^a^

nucleotide	aemplating base	*k*_cat_/*K*_m_ (μM^–1^ s^–1^)	*D*	*k*_cat_ (s^–1^)	*K*_m_ (μM)
UTP	A	2.3 ± 0.2	-	308 ± 17	130 ± 9
TU-TP	A	0.38 ± 0.1	6.1	22 ± 4	58 ± 11

a Rates were determined by global fitting of presteady-state kinetic data using KinTek Explorer.^19,20^ Steady-state rate constants (*k*_cat_, *K*_m_, *k*_cat_/*K*_m_) and associated errors were calculated using eqs 1–3. Discrimination (*D*) is the ratio of *k*_cat_/*K*_m_ values from the analog versus the canonical base (eq 4). ND: not determined. Values for UTP incorporation data are from Dangerfield et al.^21^

To investigate the fate of **TU**-TP post incorporation, we enzymatically synthesized double-stranded RNA with **TU** at the 3′ end of the primer strand, pre-equilibrated it with RdRp, and then initiated extension with 100 μM of all four NTPs (Figure 3d). A single **TU** incorporation produced multiple pause sites, with only ∼25% of the RNA reaching the 40 nt full-length product, while ∼40% was trapped as persistent, nonextendable intermediates (22–38 nt long) (Figure 3e). In contrast, a normal 3′-terminal uridine yielded 66% full-length product and only 6% intermediates. These results demonstrate that **TU** incorporation induces prematurely terminated intermediates consistent with delayed chain termination, possibly leading to dissociation of the duplex RNA. The multiple pause sites suggest a mechanism where the sulfur in the ribose analog perturbs the phosphodiester backbone to inhibit replication. Future cryoEM studies will explore the structural basis for inhibition. Previous work by Kim et al. suggested **TU** causes abortive transcript formation, but in that paper such putative intermediates were only observed after 30 min of incubation at 37 °C using relatively inactive enzyme, calling into question the validity of the authors conclusions regarding intermediates as opposed to rare byproducts.^7^ In contrast, we observed inhibition and accumulation of abortive intermediates during a single enzyme turnover within seconds, revealing **TU** as a rapid and potent inhibitor of RNA synthesis.

Nucleoside analog efficacy depends on resistance to removal by the SARS-CoV-2 NSP10/14 exonuclease complex, which is unique among RNA viruses. We assessed excision efficiency using presteady-state experiments with purified NSP10/14 and dsRNA containing either 4′-thiouridine monophosphate (**TU**-MP) or uridine monophosphate (UMP) at the 3′ terminus of the primer strand (Figure 3f,g). Processive exonuclease reactions were fit to the models in Figure 3h,i using KinTek Explorer,^19,20^ and error limits were estimated by confidence contour analysis (Supporting Information Figure S5). Both reactions were best fit to a mechanism with reversible chemistry and slow NMP release. UMP was rapidly excised, with a forward chemistry rate (*k*_2_) of 231 s^–1^, while **TU**-MP was markedly slower (63 s^–1^), as reflected in the reduced amplitude and rate of decay of the 21 nt species (Figure 3f). Notably, the thio modification accelerated the rate of product release (*k*_3_) by ∼30%, thereby shortening the lag time leading to accumulation of downstream products. Although **TU** reduced the rate of excision and affinity for RNA binding to NSP10/14 (Supporting Information Figure S5c), the calculated *k*_cat_ values for **TU**-MP and UMP excision were similar, showing that **TU**-MP does not significantly inhibit the exonuclease (Table 6).

**Table 6:** Kinetic Parameters for Nucleotide Excision by SARS-CoV-2 NSP10/14^a^

dsRNA terminal nucleotide-3′	analog	*k*_cat_ (s^–1^)	*k*_chemistry_ (s^–1^)	*k*_NMP‑release_ (s^–1^)
uridine-MP21	-	21 ± 3	231 ± 30	29 ± 3
TU-MP21	UMP	18 ± 3	63 ± 8	40 ± 5

a Rates were determined by global fitting of pre-steady-state kinetic data using KinTek Explorer.^19,20^ Parameters Include *K*_cat_, *K*_chemistry_ (*k*_2_), and *k*_NMP‑release_ (*k*_3_).

### In Vitro Disposition Characteristics

Both prodrug *iBu* **TU** and nucleotide **TU** demonstrated poor passive absorptive permeability (*P*_app_) in Ralph Russ canine kidney (RRCK) cells,^24^ which is consistent with their low lipophilicity (log *D*) as determined by the shake flask log *D* measurement (Table 7).^25^ Ester prodrug *iBu* **TU** was readily hydrolyzed to **TU** (half-life (*t*_1/2_) ∼4.29–24.4 min) in plasma from preclinical species (rat and monkey) and human. **TU** was stable in both human plasma and human liver microsomes systems for at least 4 h, indicating excellent stability.

**Table 7:** Physiochemical Characteristics and Plasma Stability

compound	^a^log *D*	^b^mean *P*_app_ 10^–6^ cm/s	^c^human plasma *t*_1/2_ (min)	^c^rat plasma *t*_1/2_ (min)	^c^monkey plasma *t*_1/2_ (min)
*iBu* **TU**	0.21	0.78	10.6	24.4	4.29
**TU**	–1.49	<0.10	>240^d^	ND	ND

a Octanol/water shake flask log *D*, pH 7.4.

b Passive permeability measured in Russ Ralph canine kidney cells.

c Cross species plasma stability (1 μM test concentration).

d 10 μM test concentration ND = not determined.

Following purified enzyme incubations, both carboxylesterases (CES)­1 and 2 isoforms were shown to catalyze *iBu* **TU** hydrolysis to **TU** with *t*_1/2_ values ranging from 11.8 min (CES1) to 30.5 min (CES2). Consistent with its physiochemical attributes, nucleoside analog **TU** exhibited low plasma protein binding across species, with unbound fractions (*f*_u, p_) values of 0.60 in rats, 0.59 in dogs, 0.63 in monkeys, and 0.74 in humans. Inhibition of major human CYP isoforms by *iBu* **TU** and **TU** was examined in NADPH-supplemented human liver microsomes.^26^ Both *iBu* **TU** and **TU** demonstrated no reversible (IC_50_s > 30 μM) inhibition of CYP1A2 (phenacetin *O*-dealkylation), CYP2C8 (amodiaquine *N*-dealkylation), CYP2C9 (diclofenac 4′-hydroxylation), CYP2D6 (dextromethorphan-*O*-demethylation), and CYP3A4/5 (midazolam-1′-hydroxylation) catalytic activities.

### Preclinical Pharmacokinetics

The in vivo pharmacokinetics (PK) of **TU** were evaluated in rats and monkeys following intravenous and oral administration in both parent (**TU**) as well as prodrug (*iBu* **TU**) form (Table 8). After intravenous dosing of **TU**, plasma clearance (CL_p_) was low in rats (0.88 mL/min/kg) and monkeys (1.9 mL/min/kg) resulting in long terminal elimination *t*_1/2_ values (rats ∼21.4 h, monkeys ∼25.0 h). Steady state distribution volumes (Vd_ss_) of **TU** were in the moderate (1.53 L/kg (rats), 2.8 L/kg (monkeys)) range. The percentage of the intravenous dose excreted as unchanged **TU** in urine from rats and monkeys over a 24 h period was ∼49% and ∼8.5%, respectively, resulting in renal clearance (CL_renal_) values of 0.45 mL/min/kg and 0.12 mL/min/kg in the two species. As such, based on the Extended Clearance Classification System (ECCS),^27^ **TU** is anticipated to be cleared via renal elimination, which is also consistent with available disposition data on marketed nucleoside-based therapeutics.^28,29^ Following oral (po) administration of the parent nucleoside **TU** (10 mg/kg) as a solution in normal saline to rats and monkeys, oral absorption was rapid (*T*_max_ ∼ 0.83 h) in rats and moderate (*T*_max_ ∼ 4.5 h) in monkeys, and resulted in oral bioavailability values of 39% and 42%, respectively. The corresponding oral fraction of the dose absorbed (*F*_a_ × *F*_g_) was estimated at 40–44% across the species.

**Table 8:** In Vivo Pharmacokinetic Profile in Preclinical Species for **TU** ^a^

species	dose (mg/kg)	*C*_max_ (ng/mL)	*T*_max_ (h)	AUC_0–∞_ (ng h/mL)	CL_p_ (mL/min/kg)	CL_renal_ (mL/min/kg)	Vd_ss_ (L/kg)	*T*_1/2_ (h)	oral *F* (%)
rat	1 (iv) **TU** ^b^ ^,^ ^c^	-	-	19,600 ± 4100	0.88 ± 0.18	0.45 ± 0.09	1.53 ± 0.24	21.4 ± 4.8	-
	10 (po) **TU** ^b^	3520 ± 52.9	0.8 ± 0.3	76,800 ± 7710	-	-	-	15 ± 1.5	39 ± 4.0
	10 (po) **TU** (administered as *iBu* **TU**)^d^	1210 ± 167	1.7 ± 1.1	45,100 ± 2420	-	-	-	25.4 ± 2.2	29.2
monkey	1 (iv) **TU** ^b^	-	-	9100	1.90	0.170	2.8	25.0	-
	10 (po) **TU** ^b^	1630	4.5	38,300	-	-	-	25.0	42
	10 (po) **TU** (administered as *iBu* **TU**)^d^	2820	3	64,400	-	-	-	19.9	90

a All experiments involving animals were conducted in AAALAC-accredited facilities and were reviewed and approved by Pfizer Institutional Animal Care and Use Committee. Pharmacokinetic parameters were calculated from plasma concentration–time data and are reported as mean ± SD for *n* = 3 and mean values for *n* = 2. All pharmacokinetic studies were conducted in males of each species (Wistar rats and cynomolgus monkey).

b **TU** was administered as a solution in a normal saline (0.9% sodium chloride in water).

c Sampling time may not have been sufficient to properly characterize *t*_1/2_ and hence the reported *t*_1/2_ is approximate.

d *iBu* **TU** was administered as a solution in a normal saline. The stated dose (10 mg/kg) refers to the nominal prodrug dose, which corresponds to 7.88 mg/kg of TU.

Administration of the ester prodrug *iBu* **TU** (10 mg/kg) orally as a solution in normal saline to rats and monkeys demonstrated rapid (*T*_max_ ∼ 1.7 h) to moderate appearance (*T*_max_ ∼ 3 h) of the active nucleoside **TU** in rats and monkeys, respectively. The corresponding oral F values of **TU**, when administered as the corresponding prodrug form *iBu* **TU**, in rats and monkeys were 29.2% and 90%, respectively.

#### Efficacy of *iBu*TU against SARS-CoV-2 in Syrian Hamsters

The in vivo efficacy of *iBu* **TU** was assessed in a Syrian hamster infection model following oral administration (as 100 mg/kg twice daily (BID) or 300, 100, and 30 mg/kg, once daily (QD)) (Figure 4). In this study, treatment of infected hamsters with 100 mg/kg dose (oral, BID) resulted in 4.5 log_10_ reduction in viral RNA copies/mg lung tissue compared to the corresponding vehicle control (Figure 5A). Interestingly, complete reduction in infectious virus titers was observed in the lungs of most of the animals treated with the 100 mg/kg BID dose (Figure 5B). For the once daily treatment regimen, a dose response efficacy was observed where the 30, 100, 300 mg/kg doses resulted in 1.7, 3.9, and 4.2 log_10_ reduction in viral RNA copies/mg lung tissue, respectively, compared to the corresponding vehicle control (Figure 5A). A similar pattern was observed for the reduction of infectious virus titers in the lungs with 0.9, 5, and 4 log_10_ reduction in TCID_50_/mg lung tissue for the 30, 100, 300 mg/kg doses, respectively (Figure 5B). Molnupiravir, at 200 mg/kg BID, was included in this study as a reference control group where it showed reduction of viral RNA loads and infectious virus titers loads by 1.5 and 2 log_10_, respectively, compared to the corresponding vehicle group (Figure 5A,B). Significant reductions in lung histopathology scores were also observed in all the *iBu* **TU**-treated groups compared to the vehicle treated control (median cumulative score of 4.5 for the *iBu* **TU** vehicle group and 5 for the molnupiravir vehicle group) (Figure 5C). The *iBu* **TU** 100 mg/kg BID dose and the 100 and 300 mg/kg QD doses showed a median cumulative lung pathology score of 1.5, similar to the score of lungs from uninfected hamsters (Figure 5C). Although nonsignificant, molnupiravir at 200 mg/kg BID resulted in marked improvement in lung pathology with a median cumulative score of 2.75 (Figure 5C). Overall, treatments were generally well tolerated with no observed side effects or weight loss in any of the treated groups (Figure 5D).

**Figure 4:**
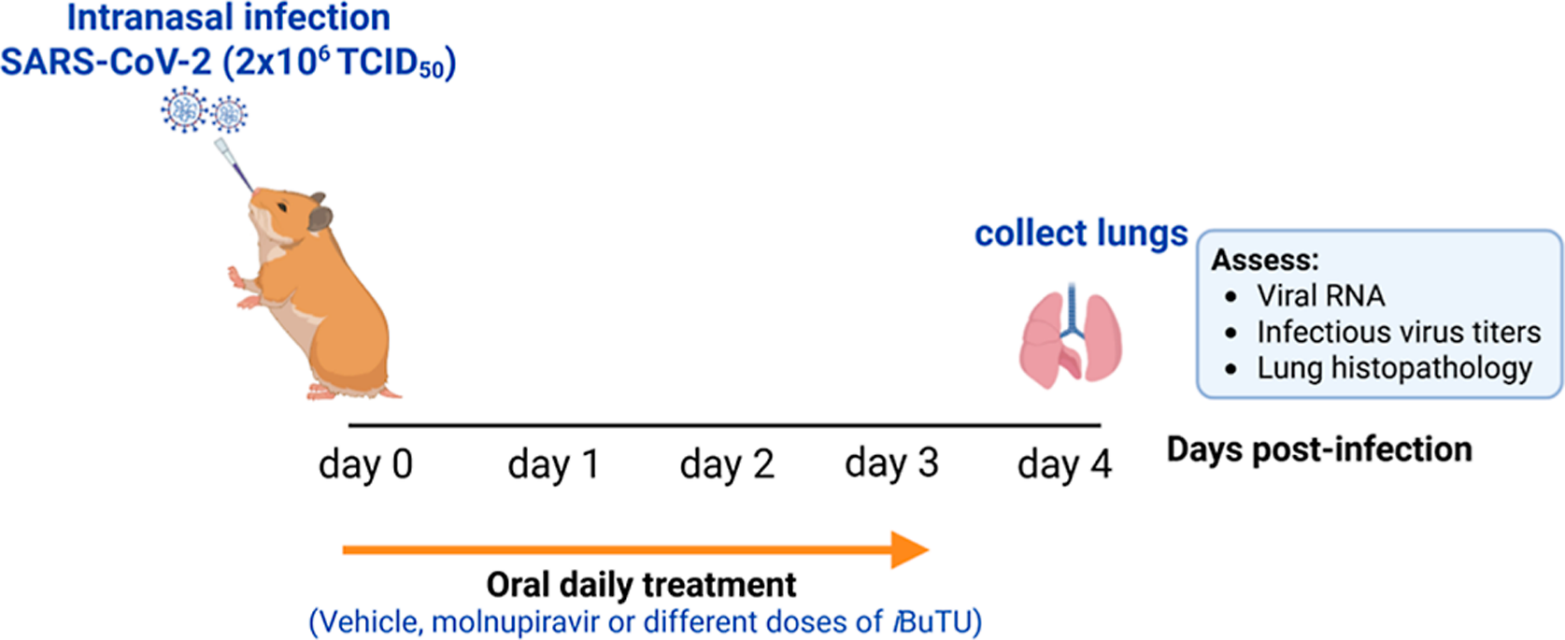
Experimental design for in vivo evaluation of *iBu* **TU** in SARS-CoV-2 infected Syrian hamsters. Animals are infected intranasally with 2 × 10^6^ TCID_50_ of SARS-CoV-2 and treated with vehicle, molnupiravir (200 mg/kg, BID) as a reference antiviral or different doses of *iBu* **TU** (orally QD or BID) for 4 consecutive days starting from the day of infection (day 0). At day 4 postinfection (pi), animals were euthanized, and lungs were collected to quantify viral loads and lung histopathology. Figure was created by BioRender.

**Figure 5:**
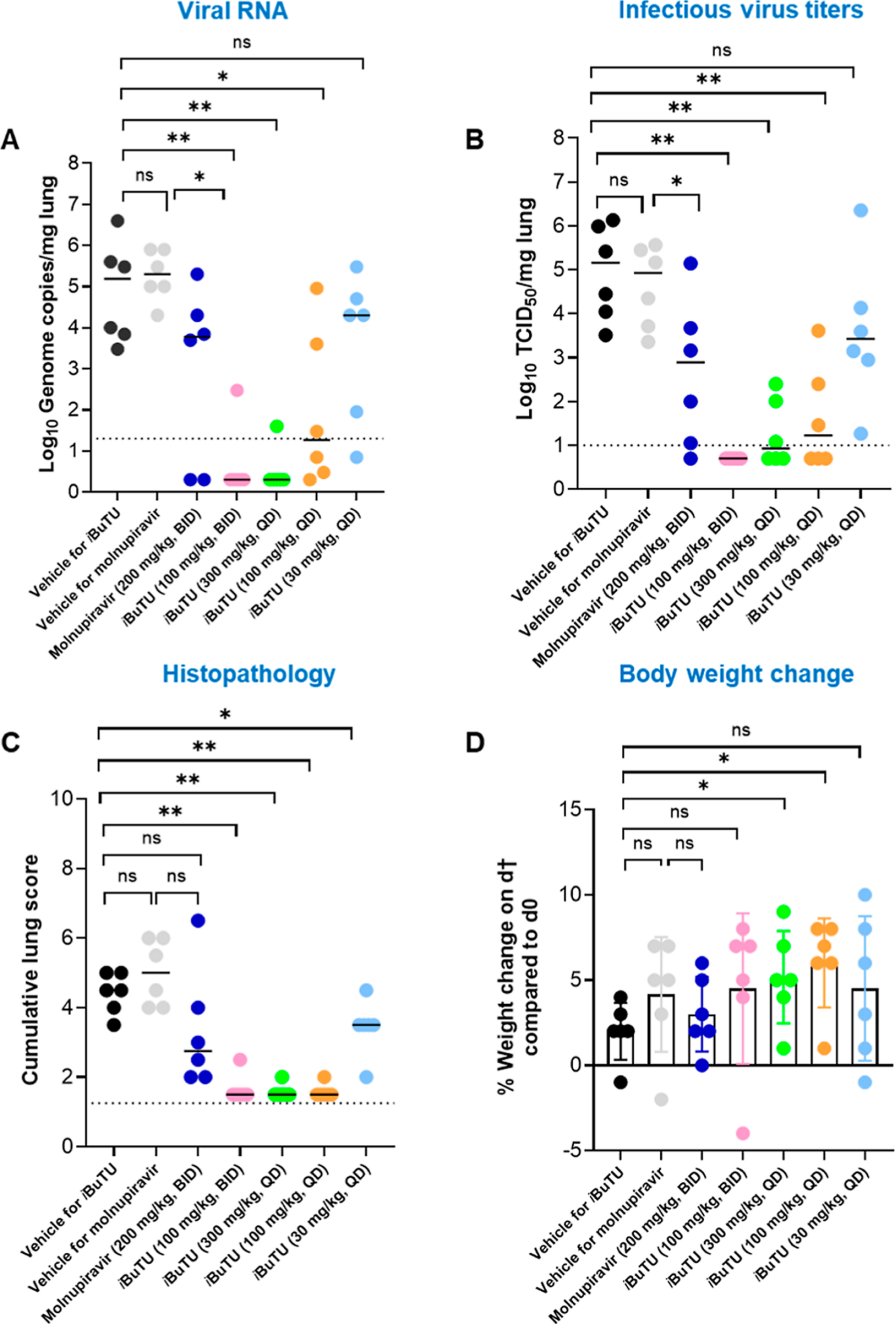
Oral efficacy of *iBu* **TU** in SARS-CoV-2 infected Syrian Hamsters. (A) Viral RNA levels in the lungs of control (vehicle-treated, BID, PO), molnupiravir (200 mg/kg, BID, PO) and *iBu* **TU**-treated (100 mg/kg, BID or 30, 100, 300 mg/kg, QD, PO) hamsters infected with 2 × 10^6^ TCID_50_ SARS-CoV-2 at day 4 pi are expressed as log_10_ SARS-CoV-2 RNA genome copies per mg lung tissue. Individual data and median values shown. (B) Infectious viral loads in the lungs of control (vehicle-treated), molnupiravir, or *iBu* **TU**-treated hamsters infected with SARS-CoV-2 at day 4 pi are expressed as log_10_ TCID_50_ per mg lung tissue. Individual data and median values are presented. (C) Cumulative severity score from H&E-stained slides of lungs from control (vehicle-treated), molnupiravir, or *iBu* **TU**-treated SARS-CoV-2-infected hamsters. Individual data and median values are presented, and the dotted line represents the median score of untreated noninfected hamsters. (D) Weight change at day 4 pi in percentage, normalized to the body weight at the time of infection (day 0). Bars represent means ± SD. All data were analyzed with the Mann–Whitney *U* test. *P < 0.05, **P < 0.01, ns = nonsignificant. Number of animals per group = 6, QD = once daily, BID = twice daily, PO = oral administration. BID dosing was administered on an 8 h schedule.

## Conclusion

Although the in vitro and in vivo profiling of **TU** was recently reported,^7^ our study provides a substantially broader and more comprehensive characterization of both **TU** and its butyryl derivative, *iBu* **TU**. Thus, both compounds were found to be potent inhibitors of SARS-CoV-2 replication in, not only Vero cells as previously described,^7^ but also in human Calu-3 and Caco-2 cells and more importantly, in primary human respiratory 3D culture models. Additionally, the safety profile of **TU** was further assessed through human primary cells and fast replicating cell-based assays and mitochondria-specific toxicity assays; all indicating a low risk of toxicity. Extensive off-target screening revealed that both **TU** and *iBu* **TU** showed no evidence of chromosomal damage and exhibited minimal interactions with a broad panel of ion channels, GPCRs, and enzymes, further supporting their favorable safety profile. Intracellularly, **TU** is metabolized to its active 5′-triphosphate form (**TU**-TP), with high concentrations observed in HAE, Vero, and Calu-3 cells, and lower levels detected in Caco-2 cells. Once formed, **TU**-TP can be incorporated into viral RNA, resulting in prematurely terminated intermediates and functioning as a delayed chain terminator to efficiently inhibit replication. The ester prodrug *iBu* **TU** is rapidly hydrolyzed by esterases to **TU** in rat, monkey, and human plasma. In contrast, **TU** itself remains stable for at least 4 h in both human plasma and human liver microsome systems, indicating excellent metabolic stability. *iBu* **TU** and **TU** demonstrated no reversible inhibition of major human CYP isoforms, derisking potential drug–drug interactions. The in vivo pharmacokinetic profiles of **TU** and its prodrug *iBu* **TU** were evaluated in both rats and monkeys. Although both compounds exhibited generally favorable characteristics, *iBu* **TU** was selected for further efficacy studies due to its superior oral bioavailability (*F* = 90%) compared to the parent nucleoside **TU** (*F* = 42%) in monkeys. While the study published by Kim et al.^7^ reports the evaluation of the parent nucleoside **TU** in a mouse model using body weight loss and survival as end points, we chose to assess the prodrug *iBu* **TU** in a more relevant Syrian hamster model infected with the SARS-CoV-2, focusing on markers of SARS-CoV-2 infection, including lung viral RNA levels, infectious viral titers, and lung tissue histopathology. Thus, oral administration of *iBu* **TU**, either once daily (QD) or twice daily (BID), led to a dose-dependent reduction of 1.7 to 4.5 log_10_ in viral RNA levels and significantly improved lung histopathology scores. The treatment was well tolerated across all groups, with no observed side effects or weight loss. Given its favorable pharmacokinetic profile, including high oral bioavailability, along with excellent safety and demonstrated in vivo efficacy, *iBu* **TU** stands out as an excellent drug candidate for the treatment of SARS-CoV-2 infection.

Pharmacokinetics studies. All procedures performed on animals were in accordance with regulations and established guidelines and were reviewed and approved by an Institutional Animal Care and Use Committee or through an ethical review process.

## Supplementary Material



## Notes

General supplements containing Scheme S1, Figures S1–S5, Tables S1–S3, NMR spectra for compounds *iBu* **TU** along with detailed protocols for preclinical pharmacokinetics studies and in vitro and in vivo antiviral evaluation (PDF)

## Abbreviations

ALI: air–liquid interface
AUC_0**–**∞_: area under the plasma concentration–time curve from time 0 extrapolated to infinity
CES: carboxylesterase; scaled intrinsic clearance
CL_p_: plasma clearance
CL_renal_: renal clearance
*C*_max_: maximum plasma concentration
*F*: oral bioavailability
*F*_a_ × *F*_g_: fraction of the oral dose absorbed
HBTECs: normal human bronchial/tracheal epithelial cells
iv: intravenous
log *D*: logarithm of the distribution coefficient
MOI: multiplicity of infection
NMP: nucleoside monophosphate
NTP: nucleoside triphosphate
PBM: primary human peripheral blood mononuclear cells
pi: postinfection
PK: pharmacokinetics
po: oral
RdRp: RNA-dependent RNA polymerase
SARS-CoV-2: severe acute respiratory syndrome coronavirus-2
SD: standard deviation
*t*_1/2_: half-life
*T*_max_: time to reach *C*_max_
Vd_ss_: steady state distribution volume.
