# Supplementary material for: The 5′-Isobutyryl Ester Prodrug of 4′-Thiouridine Inhibits SARS-CoV‑2 Replication in Culture and in a Syrian Hamster Infection Model
Source: ACS Omega. 2026 Jul 10;11(29):43231–41. doi: 10.1021/acsomega.6c00102 (PMC13425281; doi:10.1021/acsomega.6c00102)
Supplement: Supplementary file 1 [file ao6c00102_si_001.pdf]

Supporting Information

The 5'-isobutyryl ester prodrug of 4'-thiouridine inhibits SARS-CoV-2 replication in culture and in a Syrian hamster infection model

Zhe Chen[1], Franck Amblard[1]*, Mahesh Kasthuri[1], Zahira Tber[1], Julia C. LeCher[1], Sijia Tao[1], Ramyani De[1], Ingrid Marko[4], Leda Bassit[1], Keivan Zandi[1], Tamara McBrayer[1], Selwyn H. Hurwitz,[1] Birgit Weynand[5], Rana Abdelnabi,[2,3] Johan Neyts[2,3], Siddhartha Bhatt[6], Britton Boras[7], Rhonda D. Cardin[8], Yuao Zhu[8], Mili Kapoor[8], Heather Eng[6], Amit S. Kalgutkar[9], Siennah Greenfield[9], Rhishikesh Thakare[9], Amanda King-Ahmad[6], Nadini C. Patel,[9] Kenneth A. Johnson[4], David Hepworth[9], Andrew Fensome[9], Raymond F. Schinazi[1]*

[1]Center for ViroScience and Cure, Laboratory of Biochemical Pharmacology, Department of Pediatrics, Emory University School of Medicine, and Children's Healthcare of Atlanta, Atlanta, GA 30322, USA.

[2] KU Leuven, Department of Microbiology, Immunology and Transplantation, Rega Institute, Virology, Antiviral Drug & Vaccine Research Group, Leuven, Belgium.

[3]KU Leuven - Department of Microbiology, Immunology and Transplantation, VirusBank Platform, Leuven, Belgium.

[4]Department of Molecular Biosciences, University of Texas at Austin, Austin, TX 78712, USA.

[5]KU Leuven Department of Imaging and Pathology, Translational Cell and Tissue Research, Division of Translational Cell and Tissue Research, Leuven, Belgium.

[6]Research and Development, Pfizer, Groton, CT 06340, USA.

[7]Research and Development, Pfizer, La Jolla, California 92121, USA

[8]Research and Development, Pfizer, Pearl River, NY 10965, USA.

[9]Research and Development, Pfizer, Cambridge, MA 02139, USA.

***Corresponding authors,**

E-mail addresses: famblar@emory.edu (F. Amblard.), rschina@emory.edu (R. F. Schinazi).

Table of Contents:

1) Scheme S1: Synthesis of *iBu*TU S4

2) Figure S1-3: NMR spectra, and UPLC traces for *iBu*TU S6

3) Figure S4. Confidence contour analysis of kinetic data fitting for 4'-thio uridine triphosphate incorporation S9

4) Figure S5. Confidence contour analysis for nsp10/14 excision kinetics. S11

5) Table S1. Oligonucleotide Sequences. S13

6) Table S2. Secondary pharmacology assays for *iBu*TU and TU. S16

7) Table S3. Kinase cross-reactivity data for *iBu*TU and TU. S17

8) Preclinical Pharmacokinetics Studies. S18

9) In vitro and in vivo antiviral evaluation S22

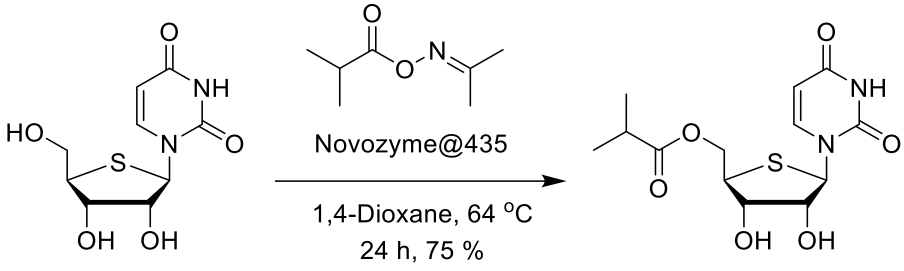

Scheme S1: Synthesis of *iBu*TU

Synthesis of ((2*R*,3*S*,4*R*,5*R*)-5-(2,4-dioxo-3,4-dihydropyrimidin-1(2*H*)-yl)-3,4-dihydroxytetrahydrothiophen-2-yl)methyl isobutyrate: Novozyme@435 (3.0 g) was rinsed with 1,4-dioxane (2 x 25 mL) and dried under high vacuum for 20 min. To a suspension of **TU** (2.0 g, 7.6 mmol)[36] in 1,4-dioxane (85 mL) was added Novozyme-435 and acetone oxime O-isobutyryl ester **3** (4.5 g, 31.4 mmol)[37] under nitrogen atmosphere. The resulting reaction mixture was stirred at 64 °C for 24 h. The reaction mixture was filtered, washed with 1,4-dioxane (150 mL) and concentrated under reduced pressure. The crude product was purified by flash column chromatography (methanol:dichloromethane: 0 to 5%) to give ***iBu*TU** as a white solid (1.89 g, 75 %). ^{1}H-NMR (400 MHz, MeOH-d_4) δ 8.04 (d, J = 8.1 Hz, 1H), 6.07 (d, J = 6.1 Hz, 1H), 5.79 (d, J = 8.0 Hz, 1H), 4.41 (m, 1H), 4.32 (m, 1H), 4.28 (m, 1H), 4.13 (t, J = 3.9 Hz, 1H), 3.59 (m, 1H), 2.63 (q, J = 7.0 Hz, 1H), 1.21 (s, 3H), 1.19 (s, 3H). ^{13}C-NMR (400 MHz, MeOH-d_4) δ 176.78, 164.5, 151.36, 141.61, 101.77, 77.06, 73.42, 64.79, 63.75, 48.90, 33.74, 17.92, 17.89. HRMS (ESI): m/z $[M+H]^+$ calcd. For $C_{13}H_{18}N_2O_6S$: 330.0886, Found: 331.0952.

***iBu*TU** was analyzed for purity by reversed-phase HPLC using a preparative-scale column: Gemini C18 (Phenomenex, 5 µm, 110 Å, 100 × 30 mm). Mobile phase: A = H_2O; B = CH_3CN.

Gradient: 0–1 min, 10% B; 1–10 min, 10 → 70% B; 10–16 min, 70 → 90% B; 16–18 min, 90 → 10% B (column wash); 18–20 min, 10% B (re-equilibration). Flow rate: 10 mL/min; column temperature: 25 °C; UV detection at 254 nm. The sample was dissolved in H_2O/CH_3CN (1:1, v/v), filtered through a 0.45 μm PTFE membrane, and injected. Chromatograms were recorded to assess purity (no collection was performed, as this method was used for analysis only). The purity of **iBuTU** was confirmed to be 96% by analytical RP-HPLC (C18 column, H_2O/CH_3CN gradient, 1.0 mL/min, UV 254 nm).

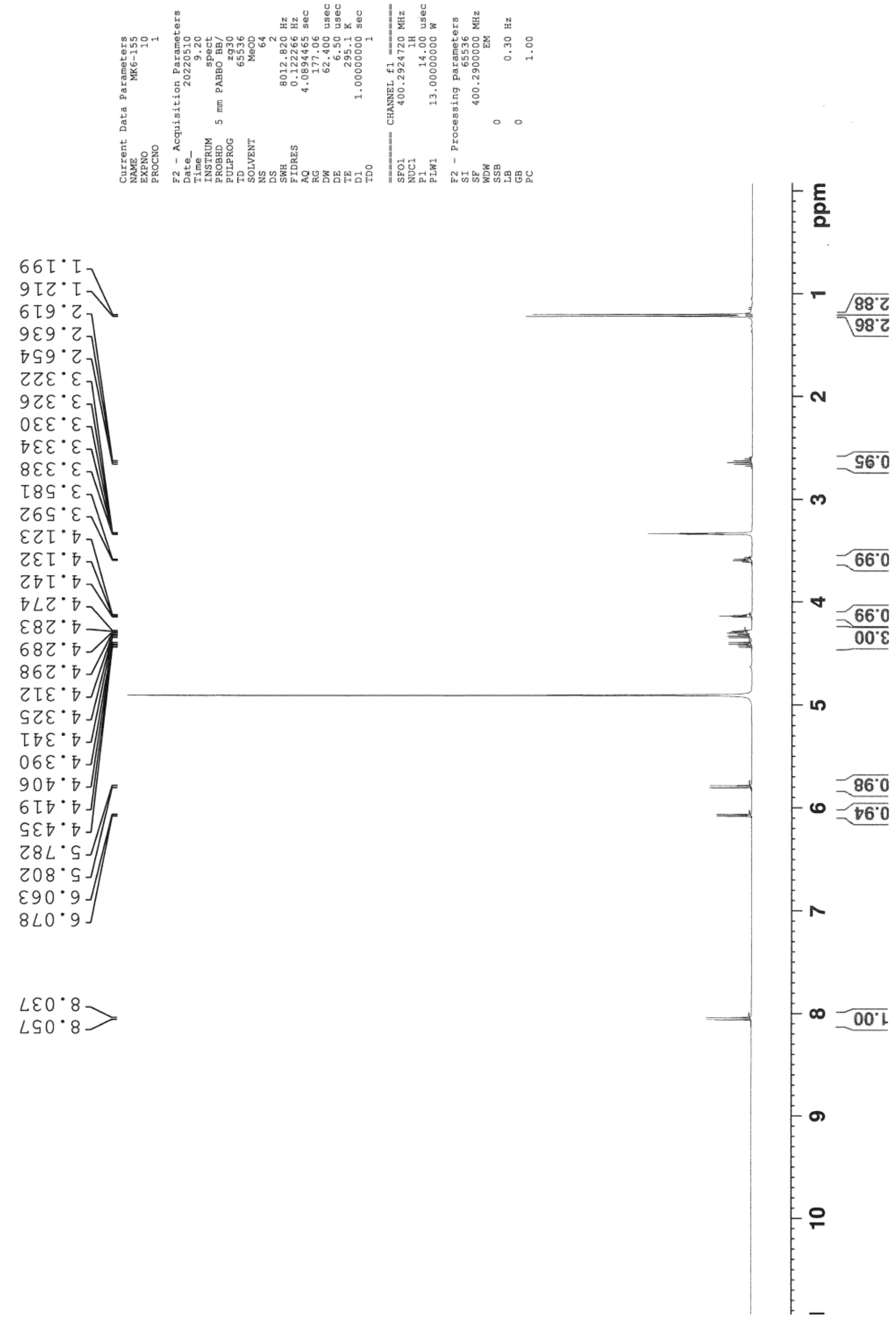

Figure S1 ^{1}H NMR spectra of *iBu*TU

.

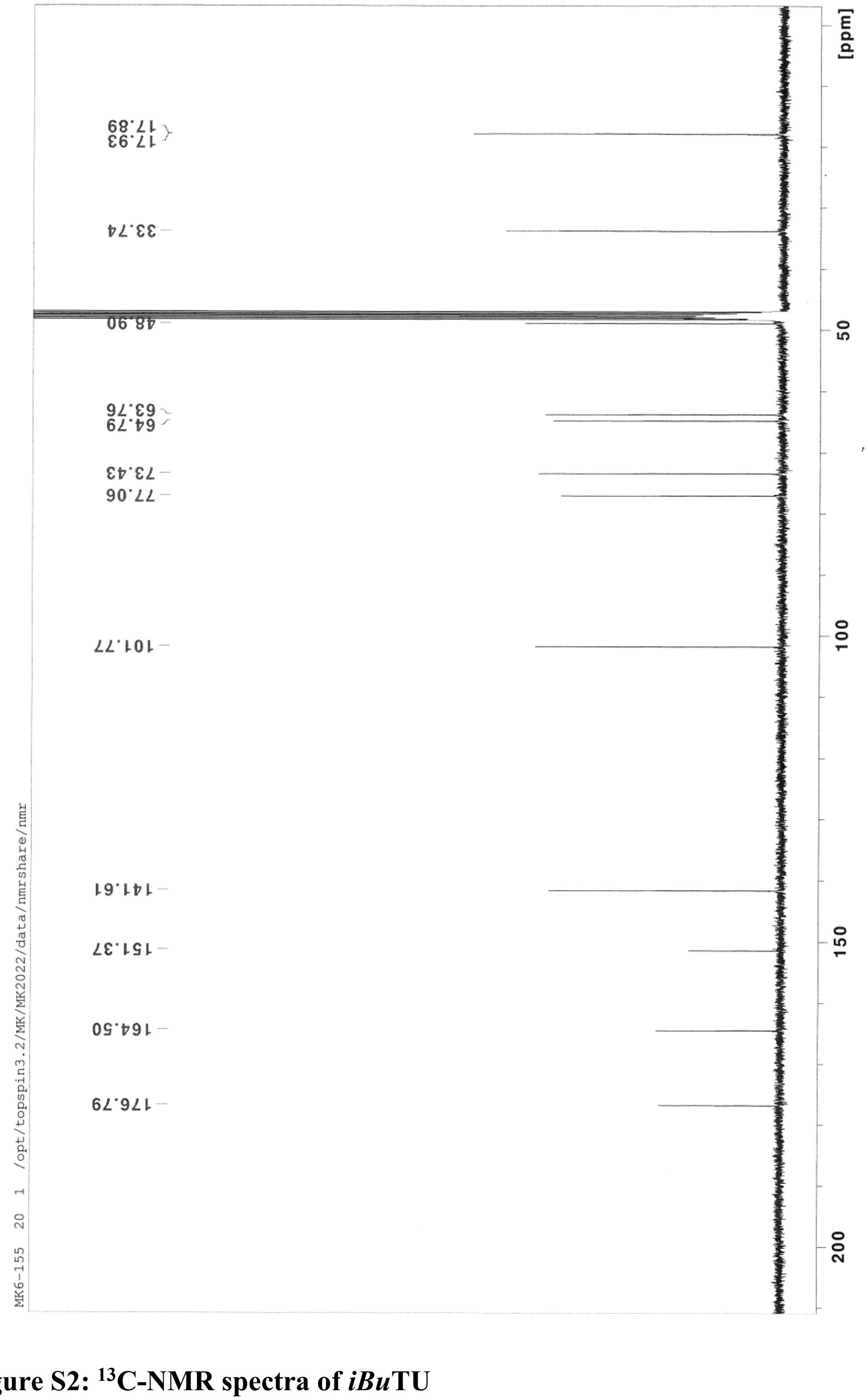

Figure S2: ^{13}C-NMR spectra of *iBu*TU

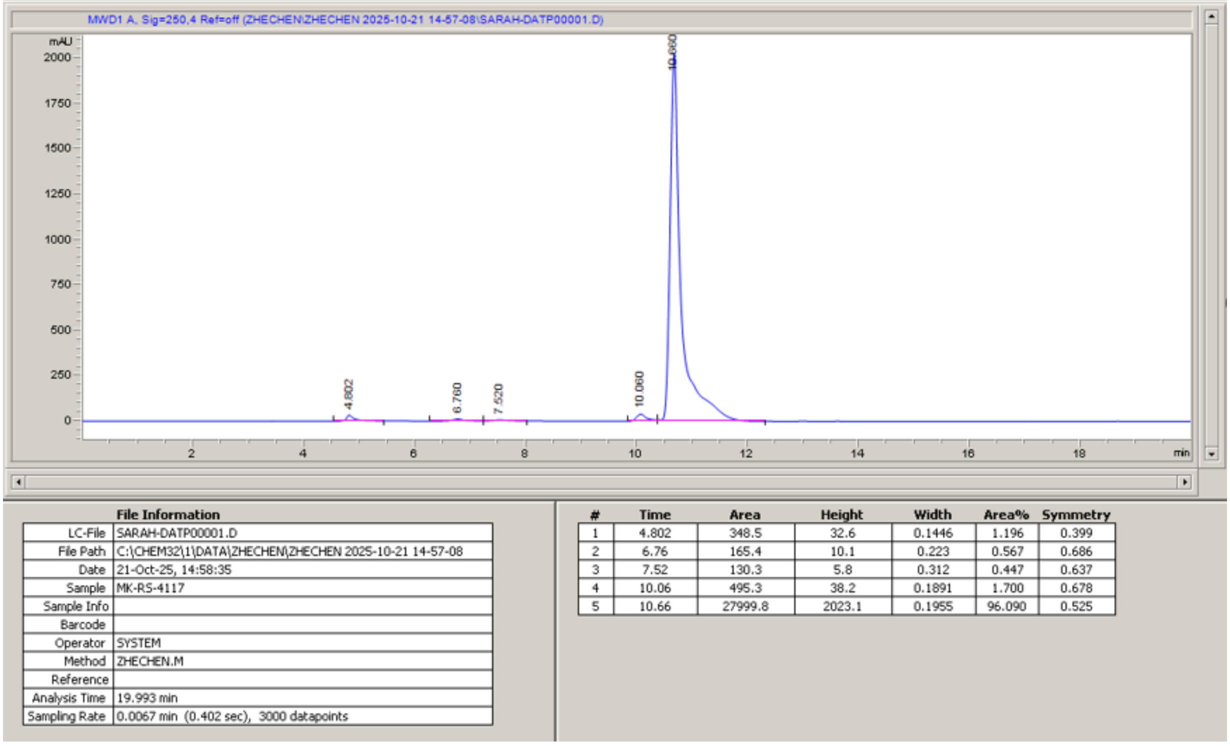

File Information

LC-File	SARAH-DATP00001.D
File Path	C:\CHEM32\1\DATA\ZHECHEN\ZHECHEN 2025-10-21 14-57-08
Date	21-Oct-25, 14:58:35
Sample	MK-RS-4117
Sample Info	
Barcode	
Operator	SYSTEM
Method	ZHECHEN.M
Reference	
Analysis Time	19.993 min
Sampling Rate	0.0067 min (0.402 sec), 3000 datapoints

#	Time	Area	Height	Width	Area%	Symmetry
1	4.802	348.5	32.6	0.1446	1.196	0.399
2	6.76	165.4	10.1	0.223	0.567	0.686
3	7.52	130.3	5.8	0.312	0.447	0.637
4	10.06	495.3	38.2	0.1891	1.700	0.678
5	10.66	27999.8	2023.1	0.1955	96.090	0.525

Figure S3: UPLC traces for *iBu*TU

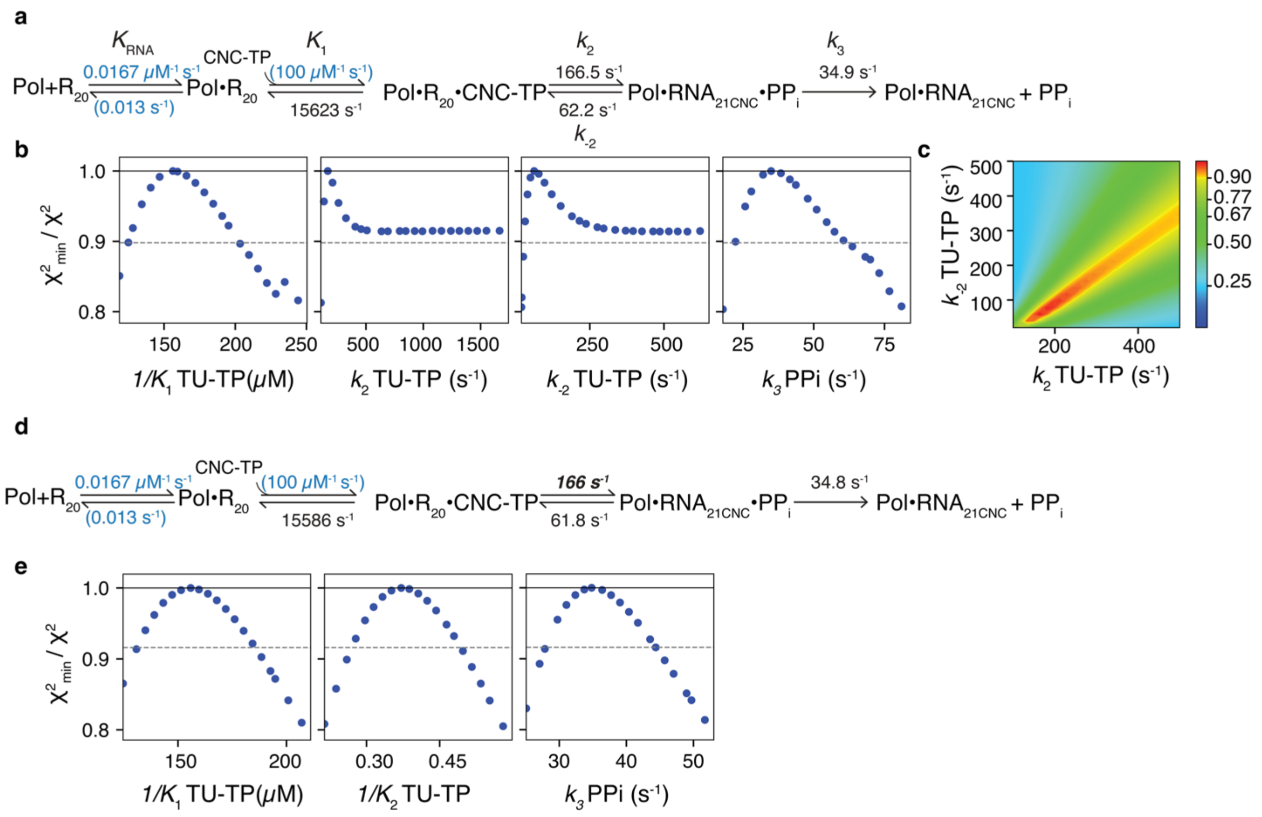

f

NSP 12/7/8 kinetic parameters for incorporation of TU-TP.

Fitting	$1/K_1$ (μM)	k_2 (s^{-1})	k_{-2} (s^{-1})	k_3 (s^{-1})
case 1	156 *(125—195)*	166*(>126)*	62 *(>30)*	35 *(22—60)*
case 2	156 *(135—184)*	166	62 *(46—80)*	35 *(30—44)*

Figure S4. Confidence contour analysis of kinetic data fitting for 4'-thio uridine triphosphate incorporation. Confidence contour analysis was performed in KinTek Explorer [19,20] to evaluate confidence intervals for parameters including K_1 (nucleotide binding, where $K_{d,\ apparent} = 1/K_1$), k_2 (forward rate of chemistry), k_{-2} (reverse rate of chemistry), and k_3 (PP_i release). Under single-turnover conditions, product rebinding was assigned as negligible ($k_{-3} = 0$) due to the low concentrations of PP_i formed during the experiment. The RNA off rate, determined by Dangerfield *et a*l. [21] was used to estimate the K_d for RNA binding in fitting the amplitudes. (a) Model for TU-TP incorporation by the RdRp, including the RNA binding to the polymerase complex (pol). Parameters fixed during fitting are shown in blue. All other rate constants were determined by

fitting by simulation. Two methods of data fitting are shown. Case 1: The rate constants for chemistry (k_2 and k_{-2}) were allowed to vary to give the confidence contours shown (b). In this case k_2 and k_{-2} have a defined lower limit and clear best fit values, but upper limits are poorly constrained. (c) 2D contour plot of k_{-2} vs. k_2 showing their ratio ($1/K_2$) is well defined. The yellow boundary marks the χ^2 threshold, gradient scale bar at the right. (d) Case 2: The rate constant for TU-TP incorporation k_2 was fixed at its best fit value to give the confidence contours (e) where all remaining parameters are well constrained. (f) Summary table of best fit parameters and upper and lower limits determined by the 95% confidence intervals based on χ^2 (dashed line) for both cases (k_2 variable or fixed). Fixing k_2 does not change the best fit values but substantially improves the error estimates for k_{-2}.

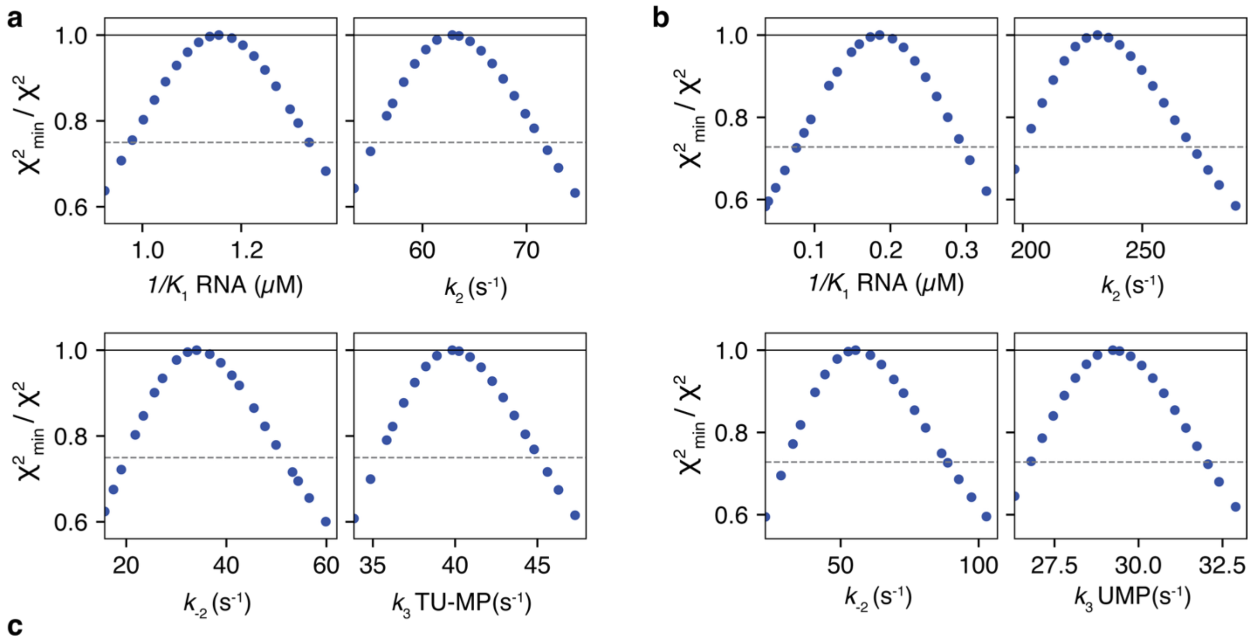

c

NSP 10/14 excision kinetic parameters

Nucleotide-3'	Analog	$1/K_1$ (μM)	k_3 (s^{-1})	k_{-3} (s^{-1})	k_4 (s^{-1})
4' thio uridine$_{21}$	*UMP*	1.1 *(0.98—1.3)*	63 *(56—71)*	34 *(22—50)*	39 *(36—45)*
uridine$_{21}$	—	0.19 *(0.09—0.29)*	231 *(204—268)*	55 *(33—87)*	29 *(27—32)*

Figure S5. **Confidence contour analysis for nsp10/14 excision kinetics.** Confidence contour analysis was performed in KinTek Explorer[19,20] to assess the goodness of fit and estimate the apparent RNA dissociation constant ($1/K_1$), the forward chemistry rate (k_2), the reverse chemistry rate (k_{-2}), and the nucleotide monophosphate (NMP) release rate (k_3). Under single-turnover conditions, product inhibition was assumed negligible (k_{-3}=0) due to the low concentration of NMP formed. (a) Confidence contour for TU-MP excision from a 21/40-nt RNA; all parameters well constrained. (b) Confidence contour for UMP excision from a 21/40-nt RNA; all parameters well constrained. (f) Summary table of the best fit parameters, with upper and lower limits determined by the 95% confidence intervals based on χ^2 (dashed line).[38]

Preparation of recombinant proteins

NSP12/7/8 co-expression

The N-terminally 6xHis tagged NSP12 was co-expressed with NSP7 and NSP8 in a pQE vector as previously described in *E. coli* BL21 electrocompetent cells with the pGTF2 chaperone plasmid[19,20] The chaperones were induced with tetracycline for 20 minutes. and NSP12/7/8 expression was induced by adding IPTG and incubation continued overnight at 16°C with shaking. Cells were harvested, and pellets were stored at −80°C until use.

NSP8 expression

The pcIts,ind+-(NSP8) plasmid was expressed as previously reported by Dangerfield et al into *E. coli* BL21 cells[20].

NSP10/14 expression

The pK27Sumo_His-SUMO-nsp10-nsp14 fusion construct (SARS-CoV-2, Addgene plasmid #169161[6]) was transformed into chemically competent E. coli T7 Express LsyY/Iq (NEB). Expression was induced by adding IPTG (0.1 mM) and incubating overnight with shaking at 16°C, as previously described[19]. Cells were harvested, and pellets were stored at −80°C until use.

Protein Purification

NSP12/7/8 and NSP10/14 protein complexes were purified as described[19]. Cell pellets were lysed with a Microfluidics LM20 microfluidizer in buffer containing 50 mM Tris pH 8, 250 mM NaCl, 30 mM imidazole, 10% glycerol, 2 mM DTT, and protease inhibitors. Clarified lysates were loaded onto a HisTrap HP column, washed with imidazole-containing buffer to remove non-specific proteins, and eluted with a linear imidazole gradient. For NSP12/7/8, pooled His-tagged fractions

were dialyzed into low-salt buffer, applied to a Heparin column, and eluted with a NaCl gradient; TEV cleavage was omitted, and pure fractions were concentrated and dialyzed into high-glycerol storage buffer (50 mM Tris pH 7.5, 50 mM NaCl, 0.1 mM EDTA, 50% glycerol, 1 mM DTT). For NSP10/14, pooled His-SUMO-tagged fractions were digested overnight with SUMO protease, dialyzed into low-salt, low-imidazole buffer, passed over a HisTrap to remove tag and uncleaved fusion, and further purified by Heparin chromatography before dialyzing into storage buffer. Protein purity was assessed by SDS-PAGE at each step, and concentrations were determined from A280 using extinction coefficients of 181,300 $M^{-1}cm^{-1}$ (NSP12/7/8) and 105,200 $M^{-1}cm^{-1}$ (NSP10/14).

Preparation of RNA substrates

RNA oligonucleotides were purchased from Integrated DNA Technologies (IDT) and RNase-free HPLC–purified, resuspended in sterile ddH_2O, and annealed in 10 mM Tris-HCl pH 7.0, 50 mM NaCl, and 0.1 mM EDTA by heating to 75 °C for 3 min followed by slow cooling over 2 hours[19,20]. Concentrations were determined from extinction coefficients (Table S1) or, for incorporated analogs, from the weighted sum of RNA species quantified by capillary electrophoresis.

Table S1. **Oligonucleotide Sequences.** Primers labeled with 6-FAM (6-carboxyfluorescein). The templating base for the next nucleotide incorporation is shown in bold and underlined.

**Oligo Name**	**Sequence (5'—3')**	**ε_{260} (M^{-1} cm^{-1})**
FAM—LS2	[6-FAM]—GUCAUUCUCCUAAGAAGCUA	222,360

FAM—LS2U	[6-FAM]—GUCAUUCUCCUAAGAAGCUAU	230,960
LS1.2A	CUAUCCCCAUGUGAUACGC**A**UAGCUUCUUAGGAGAAUGAC	397,900

Preparation of the exonuclease RNA substrates.

NSP12/7/8, 8 µM NSP8, 1 µM 6-FAM-LS2/LS1.2A, and 25 µM TU-TP (final concentrations) and were incubated at room temperature for 1 h . The mixture was heated to 60 °C for 3 min, cooled to room temperature over 1 hr, centrifuged to remove precipitated RdRp, and RNA was purified (Zymo Oligo Clean & Concentrator); composition was confirmed by capillary electrophoresis (96% TU at position 21, 4% unincorporated at position 20).

Kinetic Analysis of Polymerase and Exonuclease Activity

Kinetic assays were performed as described previously[19,20]. Reactions were performed at 37 °C using a KinTek RQF-3 and described in the detailed by Amblard et al [1]. Products were analyzed by CE with a Cy3-labeled DNA migration standard[21].

Polymerase Assays

The RdRp-RNA complex (4 µM NSP12/7/8, 8 µM NSP8, 0.1 µM FAM-LS2/LS1.2A dsRNA) in reaction buffer (40 mM Tris-HCl pH 7.0, 50 mM NaCl, and 1 mM DTT) was pre-equilibrated on ice for twenty minutes. The complex was mixed with 1.3, 2.5, 5.0, 10, 20, 30, 40, 80, 200, 300 µM TU-TP in reaction buffer plus 5mM $MgCl_2$ (after mixing).

Exonuclease Assays

NSP10/14-RNA complex (4 μM NSP10/14, 0.1 μM dsRNA) in 40 mM Tris-HCl pH 7.0, 50 mM NaCl, 2.5 mM EDTA, and 1 mM DTT was mixed with buffer containing 15 mM $MgCl_2$. Final reactions contained 2 μM NSP10/14, 0.05 μM dsRNA, 6.25 mM $MgCl_2$, 1.25 mM EDTA-Mg^{2+}, and 1 mM DTT.

Data fitting and analysis

Kinetic data were analyzed in KinTek Explorer (v11) by globally fitting to a defined mechanistic model[20] through numerical integration of rate equations. Initial parameters were estimated from standard fits (single/double exponentials, burst equation) and dynamic simulations, then refined iteratively by minimizing χ^2 with residuals normalized using sigma values. Confidence contour analysis was performed to assess parameter uncertainty and fit quality[19,38].

<u>Calculation of Steady-State parameters and other statistics</u>

Equations derived for mechanisms with reversible chemistry and slow product release[5,19,39]:

(Eq. 1) $$k_{cat} = \frac{k_2 k_3}{k_2 + k_{-2} + k_3}$$

(Eq. 2) $$K_m = \frac{k_2 k_3 + k_{-1}(k_{-2} + k_3)}{k_1(k_2 + k_{-2} + k_3)}$$

(Eq. 3) $$k_{cat}/K_m = k_1 \frac{k_2 k_3}{k_2 k_3 + k_{-1}(k_{-2} + k_3)}$$

Discrimination Index (D) between natural nucleotide (NTP) and nucleotide analog (AnaTP) [21]:

(Eq. 4) $$D = \frac{(k_{cat}/K_m)_{NTP}}{(k_{cat}/K_m)_{AnaTP}}$$

(Eq. 5) $$Nucleotides\ incorporated\ per\ analog = D \times [NTP]/[AnaTP]$$

Table S2. Secondary pharmacology assays for *iBu*TU and TU.

Structure	*iBu*TU	TU
PatchXpress hERG (HEK293)<br>IC_{50} (nM)	>100000	>100000
Nav1.5 CHO - 1 Hz, Vh=-80 mV (Qpatch)<br>IC_{50} (nM)	>100000	>100000
Dofetilide binding (CHO)<br>Ki (nM)	>92818	6859330
Alpha 1a adrenergic receptor (antagonist, CHO)<br>IC_{50} (nM)	>30000	>30000
Alpha 1a adrenergic receptor (agonist, CHO)<br>EC_{50} (nM)	>30000	>30000
Cannabinoid CB1 (antagonist, CHO)<br>IC_{50} (nM)	>30000	>30000
Cannabinoid CB1 (agonist, CHO)<br>EC_{50} (nM)	>30000	>30000
Dopamine D1 (antagonist, CHO)<br>IC_{50} (nM)	>30000	>30000
Dopamine D1 (agonist, CHO)<br>EC_{50} (nM)	>30000	>30000
Histamine H1 (antagonist, HEK-293)<br>IC_{50} (nM)	>30000	>30000
Histamine H1 (agonist, HEK-293)<br>EC_{50} (nM)	>30000	>30000
mu-opioid (antagonist, CHO)<br>IC_{50} (nM)	>30000	>30000
mu-opioid (agonist, CHO)<br>EC_{50} (nM)	>30000	>30000
Histamine 5HT-2B (agonist, CHO)<br>EC_{50} (nM)	>30000	>30000
Muscarinic M3 (antagonist, CHO)<br>IC_{50} (nM)	>30000	>30000
Muscarinic M3 (agonist, CHO)<br>EC_{50} (nM)	>30000	>30000
Phosphodiesterase 3B (SF9 cells)<br>IC_{50} (nM)	>30000	>30000
Phosphodiesterase 4D2 (SF9 cells)<br>IC_{50} (nM)	>30000	>30000
Acetyl cholinesterase (HEK-293)<br>IC_{50} (nM)	>30000	>30000

Table S3. Kinase cross-reactivity data for *iBu*TU and TU.

	*iBu*TU	TU
**ATP = K_m**		
**Aurora-A**	1.180	5.084
**BTK**	8.928	15.968
**CDK2**	9.415	8.114
**CHK1**	4.153	6.868
**CHK2**	4.112	1.887
**SRC**	12.269	13.271
**EGFR**	-3.339	-1.106
**EPHA2**	5.542	6.707
**FGFR1**	7.803	6.393
**GSK3b**	5.733	18.510
**INSR**	1.946	1.603
**IRAK4**	-0.481	-4.196
**JAK3**	-1.335	-2.746
**KDR**	6.779	5.172
**LCK**	12.779	5.838
**ERK2**	9.395	11.260
**MAPKAPK2**	-0.874	-1.363
**MARK1**	8.257	1.034
**MET**	-11.070	5.360
**MST2**	9.692	7.890
**PAK4**	0.970	-1.191
**PIM2**	5.940	10.099
**PRACa**	5.666	5.388
**AKT1**	6.812	7.479
**ROCK1**	3.377	5.236
**SGK**	5.019	0.371
**TEK**	5.150	-0.216
**TRKA**	9.812	-1.040
**TAOK2**	4.579	-1.372
**CAMK2A**	1.163	3.467
**CK1a1**	9.019	7.008
**MAP4K4**	28.715	43.304
**MST4**	17.671	11.758
**MYLK2**	7.392	5.191
**PDK1**	2.254	-0.646
**P38**	6.429	8.509
**ATP = 1 mM**		

**Aurora-A**	0.406	2.865
**BTK**	8.846	7.285
**CDK2**	0.541	1.696
**CHK1**	-0.412	1.583
**CHK2**	-3.682	-4.697
**SRC**	22.646	28.458
**EGFR**	-9.666	-2.559
**EPHA2**	8.620	-8.690
**FGFR1**	-0.022	0.496
**GSK3B**	9.470	14.165
**ISNR**	7.460	5.329
**IRAK4**	4.853	3.615
**JAK3**	-2.670	3.636
**KDR**	7.151	7.436
**LCK**	24.241	13.971
**MAPK1**	16.768	16.385
**MAPKAPK2**	-11.749	-8.519
**MARK1**	3.396	5.424
**MET**	10.065	-0.669
**STK3**	0.572	0.952
**PAK4**	2.343	4.904
**PIM2**	2.816	2.354
**PRKACA**	8.798	11.690
**AKT1**	1.234	3.177
**ROCK1**	-0.576	-1.296
**SGK**	-2.890	1.581
**TEK**	-4.401	1.395
**TRKA**	31.706	15.563
**TAOK2**	2.748	5.498

Preclinical Pharmacokinetics Studies. All activities involving animals were carried out in accordance with federal, state, local and institutional guidelines governing the use of laboratory animals in research in AAALAC accredited facilities and were reviewed and approved by Pfizer's Institutional Animal Care and Use Committee.

Rat Pharmacokinetics. Rat pharmacokinetics studies were done at Pfizer (Groton, CT); Jugular vein- and carotid artery-cannulated male Wistar-Hannover rats were purchased from Charles River Laboratories, Inc. (Wilmington, MA) and were typically 7-10 weeks of age at the time of dosing. During the pharmacokinetic studies, all animals were housed individually. Access to food and water was provided ad libitum (i.e., subjects were dosed in the fed state). TU was administered

intravenously (iv) via the jugular vein cannula (*n* = 3) dosed as a solution (1 mg/kg, 1 ml/kg). TU or iBuTU was dosed via oral (po) gavage (*n* = 3) as a solution (10 mg/kg, 10 ml/kg). All doses were administered as a solution in normal saline (0.9% sodium chloride in water). Serial blood samples were collected via the carotid artery cannula at predetermined timepoints after dosing. Urine samples (0–24 or 0-48 hr) were also collected after iv administration. At the completion of the study, animals were euthanized by overdose of inhaled anesthesia followed by exsanguination. Blood samples were collected into tubes containing K_2EDTA and stored on ice until centrifugation to obtain plasma, which was stored frozen at -20 °C or lower.

Monkey Pharmacokinetics. Monkey studies were conducted at Pfizer (Groton, CT). Male Cynomolgus monkeys were purchased from Covance (Princeton, NJ), Charles River Laboratories, Inc. (Wilmington, MA), or Envigo Global Services (Indianapolis, IN); subjects 3-8 years of age were used in pharmacokinetics studies. RS-3995 was dosed iv by the saphenous vein (1 mg/kg, 1 ml/kg, *n*=2). RS-3995 or RS-4117 was dosed via po gavage (10 mg/kg, 10 ml/kg, *n*=2). All doses were administered as a solution in normal saline (0.9% sodium chloride in water). Serial blood samples were collected via the femoral vein before dosing and at predefined time points post-dose. Urine samples (0–48 h) were also collected after iv administration. Blood samples were collected into K_3EDTA treated collection tubes and were stored on wet ice prior to being centrifuged to obtain plasma, which was stored frozen at - 20 °C or lower.

LC-MS/MS Analysis.

Urine samples were diluted 1:9 in plasma. Plasma or urine samples (20 µl) were processed using protein precipitation with 1:1 acetonitrile:methanol (150 µl) containing terfenadine (5 ng/ml) or buspirone (5 ng/ml) as an internal standard followed by quantitation against a standard curve (0.1-2500 ng/ml) prepared in blank plasma. Samples were evaporated followed by reconstitution in water containing 0.1% formic acid. Briefly, a Waters ACQUITY ultra performance liquid chromatography system (Waters, Milford, MA) coupled to a Sciex 6500 triple quadrupole mass spectrometer equipped with an electrospray ionization source was used. Chromatographic separation was accomplished using a Phenomenex Luna Omega polar C18 column (2.6 µm, 2.1 × 100 mm) maintained at 45 °C. The mobile phase (2 solvents gradient) was optimized to achieve good separation between the analytes. Solvent A constituted of 0.1% formic acid in water, and solvent B consisted of 0.1% formic acid in acetonitrile. The gradient generally began at 5-30% B

until about 1 min, followed by an increase to 95% B to 2.0 min and held for 0.5 min, then decreased to 5-30% B until ~2.6 min. MS/MS methods for the analytes were (Q1/Q3, collision energy). Analyst v.1.7 software was used for peak integration and Watson v.7.5 (Thermo Scientific, Waltham, MA) was used for standard curve regression.

Pharmacokinetic Analysis. Pharmacokinetic parameters were calculated using noncompartmental analysis (Watson v.7.5). The area under the plasma concentration-time curve from t = 0 to infinity (AUC0-∞) was estimated using the linear trapezoidal rule.

Plasma clearance (CL_p) was calculated as:

$$CL_p = \frac{Dose_{iv}}{AUC_{0-\infty}}$$

The terminal rate constant (k_{el}) was calculated by linear regression of the terminal phase of the log-linear concentration-time curve and the terminal elimination $t_{1/2}$ was calculated as:

$$t_{\frac{1}{2}} = \frac{0.693}{k_{el}}$$

Steady state distribution volume (Vd_{ss}) was determined by clearance multiplied by mean residence time. Oral bioavailability (F) was defined as:

$$F = \frac{AUC_{po} \times Dose_{iv}}{AUC_{iv} \times Dose_{po}}$$

Bioavailability of active compound after oral administration of prodrug was calculated by dividing the dose-normalized active $AUC_{0-\infty}$ after oral dosing of prodrug by the dose-normalized active $AUC_{0-\infty}$ after iv dosing of active, accounting for their respective differences in molecular weight:

$$F = \frac{\boldsymbol{TU}\ AUC_{po,prodrug} \times \boldsymbol{TU}\ Dose_{iv,ctive}}{\boldsymbol{TU}\ AUC_{iv,active} \times \boldsymbol{TU}\ Dose_{po,prodrug}}$$

where the subscripts denote the dosed entity.

The fraction of the oral dose absorbed (F_a x F_g) was estimated using the equation (Kato et al., 2003):

$$F_a \times F_g = \frac{F}{1 - \frac{CL_{blood}}{Q}}$$

A hepatic blood flow (Q) of 70 ml/min/kg and 44 ml/min/kg was used for rats and monkeys, respectively (Davies and Morris, 1993). Blood clearance (CL_{blood}) was calculated by dividing CL_p by the blood-to-plasma ratio in the respective preclinical species.

The total amount of compound excreted unchanged in urine was calculated using the following equations and summing the amount in all collection intervals, where C_u is the measured urine concentration, DF is the dilution factor applied during bioanalysis, and V_u is the volume of urine collected during each time interval.

$$Amount\ in\ Urine = C_u \times DF \times V_u$$

Renal clearance (CL_{renal}) was calculated as:

$$CL_{renal} = \frac{Amount\ in\ urine}{Amount\ dosed} \times CL_p$$

Plasma, CES, and Liver Microsome Stability Studies

Plasma. Frozen pooled plasma from Wistar Han rats (male and female), Cynomolgus monkey (male and female) and human (male and female) collected with K_2EDTA was purchased from BioIVT (Woodbury, NY). Substrates were diluted in DMSO (final concentration of DMSO in the incubations were ≤ 0.1%). Incubations were conducted at 37 °C in duplicate. Periodic aliquots (40 µl) of the incubation mixture at 1, 4, 7, 12, 25, 45, 60, and 120 min were removed and quenched in acetonitrile (160 ml) containing diclofenac (25 ng/ml) as internal standard. Sample processing and LC-MS/MS analysis are described below. The mono-exponential decline of the peak area of analyte to internal standard versus time was used to calculate $t_{1/2}$ using E-WorkBook v10 (ID Business Solutions, Guildford, Surrey, UK).

CES. Human CES1b, CES1c, and CES2 were purchased from Fisher Scientific (Waltham, MA). CES1b and CES1c were combined at a dilution of 0.25 mg/ml each in phosphate buffered saline. CES2 was diluted to 0.5 mg/ml in phosphate buffered saline. Substrates were diluted in DMSO (final concentration of DMSO in the incubations were ≤ 0.1%). Incubations were conducted at 37 °C in duplicate. Periodic aliquots (40 µl) of the incubation mixture at 1, 4, 7, 12, 25, 45, 60, and 120 min were removed and quenched in acetonitrile (160 ml) containing diclofenac (25 ng/ml) as internal standard. Sample processing and LC-MS/MS analysis are described below. The mono-exponential decline of the peak area of analyte to internal standard versus time was used to calculate $t_{1/2}$ using E-WorkBook v10 (ID Business Solutions, Guildford, Surrey, UK).

Liver microsomes. Compound (1 µM) was incubated in human (pool of 50 donors of mixed gender) liver microsomes purchased from Sekisui XenoTech (Kansas City, KS) (1 mg/ml) in potassium phosphate buffer (100 mM, pH 7.4) supplemented with $MgCl_2$ (3.3 mM) and β-Nicotinamide adenine dinucleotide phosphate, reduced form (NADPH) (1.3 mM) in a final volume of 400 µl. Stock were prepared in acetonitrile (final concentration of acetonitrile in the incubations were ≤ 0.5%). Incubations were conducted at 37 °C in triplicate. Control incubations in the absence of NADPH were also conducted in parallel in duplicate. Periodic aliquots (40 µl) of the incubation mixture at 0, 5, 10, 15, 20, 30, 45 and 60 min were removed and quenched in acetonitrile (160 ml) containing diclofenac (25 ng/ml) as internal standard.

Samples from the microsomal stability assays were vortexed, centrifuged (5 min, 2300 x g), and the supernatant was diluted with an equal volume of water. Samples were analyzed for depletion of compound by LC-MS/MS (see the method described above for pharmacokinetics samples). Analyst software (Sciex, Framingham, MA) was used to measure peak areas. Peak area ratios of analyte to internal standard were calculated. Compound depletion half-life ($t_{1/2}$) was calculated using E-WorkBook v10 (ID Business Solutions, Guildford, Surrey, UK). The natural log of peak area ratios versus time were fitted using linear regression, and the slope (k) was converted to $t_{1/2}$ values, where $t_{1/2} = -0.693/k$.

In vitro and in vivo antiviral evaluation

Cells, media, and culture conditions

Human hepatocarcinoma liver epithelial cells (Huh-7; JCRB0403) were purchased from JCRB Cell Bank, JCRB. African Green Monkey Kidney cells (Vero; ATCC® CCL-81TM) and human lymphoblasts (CEM; ATCC® CRL-2265TM) were purchased from ATCC® (Manassas, VA, USA). Primary human peripheral blood mononuclear (PBM) cells were isolated in-house from human blood (pooled 3 donors) obtained from NY Blood Bank (NY, NY, USA) as previously described.[30] Normal human bronchial/tracheal epithelial cells (HBTECs) were purchased from Lonza Biosciences (CC-2540s, Basal, Switzerland). Media compositions were as follows: (1) Huh-7 - Dulbecco's modified eagle medium (DMEM), 10% fetal bovine serum (FBS), 100 U/mL penicillin-streptomycin (pen-strep), and 2 μM L-glutamine (L-glut), (2) Vero – DMEM, 10% FBS, 100 U/mL pen-strep, and 2 μM L-glut, (3) CEM - Roswell Park Memorial Institute 1640 (RPMI 1640) medium, 10% FBS, and 100 U/100 μg/mL pen/strep, (4, 5) PBM, MDM - RPMI 1640, 10% FBS, 100 IU/mL IL-2, and 100 U/100 μg/mL pen/strep. HBTEC cells were cultured as previously described.[31,14] In brief, basal cells were expanded in complete PneumaCult™ ExPlus medium (STEMCELL™ Technologies, USA) supplemented with hydrocortisone solution (STEMCELL™ Technologies, USA). For air-liquid interface (ALI) cultures, 1.5 x 10^5 cells were seeded on a 24-well 0.4 μm polystyrene transwell insert (Corning® USA) coated with type IV human collagen type IV (Sigma, USA) in complete PneumaCult™ ExPlus medium. After 3 days, media was removed from the upper apical chamber to expose this surface to the air and complete Pneuma-Cult™-ALI Media supplemented with Pneuma-Cult™-ALI supplements (STEMCELL™ Technologies, USA), hydrocortisone (STEMCELL™ Technologies, USA), and heparin solution (STEMCELL™ Technologies, USA) added to the basal chamber to induce differentiation. Media was refreshed in the basal compartment every 2-3 days for 3-4 weeks. Culture viability and establishment of an intact polarized epithelial layer was routinely analyzed by measuring transepithelial electrical resistance (TEER; values > 250 Ω*cm^2) and through microscopic analysis of cell morphology, ciliary beat frequency, mucus formation, and the absence of barrier-leakage. Airway organoids (hBORBs) were generated from HBTECs as previously described by the Takayama Lab at Georgia Institute for Technology in Atlanta, GA.[14] In brief, ~3k HBTEC basal cells were seeded into 384-well hanging drop plates with in Pneumacult airway organoid basal media (STEMCELL™

Technologies, USA) enriched with organoid seeding supplement (STEMCELL™ Technologies, USA), methyl cellulose, and Matrigel (Corning) using a patented gel-cooling technique to produce inverted apical-out organoids. Organoids were differentiated over 21 days in Pneumacult™ Airway organoid basal media enriched with differentiation supplements (STEMCELL™ Technologies, USA) with media changes every 3-4 days. For all experiments, cells were grown at 37 °C in a 95% O_2, 5% CO_2 injected incubator.

Coronavirus strains, propagation, and titration.

The following reagents were deposited by Centers for Disease Control and Prevention and obtained through BEI Resources, NIAID, NIH: 1) SARS-Related Coronavirus 2, Isolate hCoV-19/USA-WA1/2020, NR-52281 (Washington strain, propagated in Vero cells), 2) SARS-Related Coronavirus 2, Isolate USA/CA_CDC_5574/2020, NR-54011 (Alpha, propagated in Vero cells), 3) SARS-Related Coronavirus 2, Isolate hCoV-19/South Africa/KRISP-EC-K005321/2020, NR-54008, contributed by Alex Sigal and Tulio de Oliveira (Beta, propagated in Vero cells), 4) SARS-Related Coronavirus 2, Isolate hCoV-19/Japan/TY7-503/2021 (Brazil P.1), NR-54982, contributed by National Institute of Infectious Diseases (Gamma, propagated in Vero cells), 5) SARS-Related Coronavirus 2, Isolate hCoV-19/USA/MD-HP05647/2021 NR-55672, contributed by Dr. Andrew S. Pekosz (Lineage B.1.617.2; Delta, propagated in Calu-3 cells). The Omicron variant B.1.1.529 was a kind gift from Dr. Mehul Suthar and can be obtained through BEI Resources, NIAID, NIH: SARS-Related Coronavirus 2, isolate hCoV-19/USA/GA-EHC-2811C/2021 (Lineage B.1.1.529; Omicron Variant), NR-56481, which was propagated in Calu-3 cells. All SARS-CoV-2-related virus stocks were titrated on cell lines utilized in testing (Vero, Calu-3, and Caco-2) via ELIspot assay as previously described[14]. In brief, cells were grown to ~90% confluency in 96-well plates and infected with virus stock in 10-fold dilutions for 90 m, then virus was removed and cells overlayed with 2% methyl cellulose prepared in respective base media containing 2% heat-inactivated FBS (ΔFBS). After 2 (Delta & Omicron) or 3 (Washington, Alpha, Beta, and Gamma) days, methylcellulose was removed, cells fixed, blocked, and incubated overnight with primary antibody against the SARS-CoV-2 nucleocapsid (SinoBiologicals 40143-R001) followed by a 1 h incubation with secondary antibody (HRP-conjugated

goat anti-mouse (Invitrogen 65-6120). For detection, 50 µL of True Blue™ substrate (SeraCare, 5510-0030) was added per well for 1 h. Plates were imaged on a CTL plate reader (Immunospot®; ELIspot Reader) and titers were calculated in one of two ways. For Vero cells, which form clear foci, FFU/mL was calculated as ((#foci)/(dilution*innoculation volume)). For Calu-3 and Caco-2, an adapted tissue culture infectious dose ($TCID_{50}$) was used whereby wells were scored for presence/absence of virus and $TCID_{50}$ values calculated using the Spearman & Kärber algorithm;[32] FFU/mL was then calculated as $TCID_{50}$ * 0.69. All SARS-CoV-2 infections were conducted in a BSL-3 level laboratory at Emory University in accordance with the guidelines of the 6th edition of Biosafety in Microbiological and Biomedical Laboratories and with the approval of the Emory University Environmental Health and Safety Office.

Viral inhibition assay

SARS-CoV-2 antiviral evaluation was performed as previously described in Vero, Calu-3, and Caco-2 cell lines.[30,33] In brief, cells were grown to confluency (10^5 cells) in 96-well plates. Dose-response curves were performed by treating cells with 2-fold serial dilutions (0-20 µM) of compound in respective base media containing 2% ΔFBS then infected with an equal volume of virus for a final compound concentration of 0-10 µM. In all cases, a multiplicity of infection (MOI) of 0.1 was used for Vero and MOI of 0.01 for Caco-2 and Calu-3 cell lines. Infections were carried out for 48 hr (Vero) or 72 hr (Caco-2 and Calu-3). Supernatants were collected in 150 µL RLT Buffer (Qiagen©, Hilden, Germany) for downstream RNA extraction following manufacture's protocol (RNeasy 96 extraction kit; Qiagen©, Hilden, Germany) and subsequent qRT-PCR to detect viral load. All SARS-CoV-2 infections were performed three independent times in triplicate and were carried out in a humidified incubator at 37°C with 95% O_2 and 5% CO_2 injection.

Advanced antiviral assays were also performed by dose-response assay with lead compounds against SARS-CoV-2 Washington strain in HBTEC-ALI and in airway organoids as previously described[14]. In brief, for HBTEC-ALI cultures, compounds were added at indicated dilutions to the basolateral chamber in full media. Cells were washed 3x with PBS on apical surface to remove excess mucus then infected with

50 μL of virus (MOI 1.0; as determined from Calu-3 titration) in basal media to the apical chamber for a 4 h adsorption period after which virus was removed and cells retained in ALI for an additional 3 days. HBTEC-ALI cultures were collected in 300 μL of Trizol™ Reagent and RNA extracted by phenyl-chloroform method according to manufactures' protocol (ThermoFisher Scientific, USA). For airway organoids, compounds were serially diluted (2-fold dilutions) and plated with organoids (1 per well) at 2x concentration (0-20 μM) in 50 μL of full culture media in 96 well plates using an automated liquid handler robot (Analytik Jena, CyBio FeliX) controlled by CyBio Composer software in the Takayama group at Georgia Technical University and then transferred to the BSL-3 facility at Emory University. Organoids were infected with 50 μL of SARS-CoV-2 (MOI 1.0; as determined from Calu-3 titration) in full culture media, bringing final compound dilution to 0-10 μM. After 3 days, organoids and media were directly collected in 150 μL RLT buffer and RNA extracted by RNeasy 96-well kit as above (All experiments were performed three independent times in duplicate and were carried out in a humidified incubator at 37°C with 95% O_2 and 5% CO_2 injection.

Quantitative RT-PCR assay for virus yield inhibition

Virus yield inhibition assays were performed as previously described.[30] For antiviral assays in primary systems, C_T values were calculated from replicate groups then virus yield quantified via standard curve. Median effective concentration of compounds (EC_{50}) and concentrations with a 90% inhibitory effect (EC_{90}) were calculated from resultant values by 4-parameter non-linear regression on GraphPad Prism, v9 (GraphPad Software Inc., San Diego, CA) and reported as the mean ± stander error of the mean (SEM).

Syrian hamster SARS-CoV-2 model: [34]

Female (6-8 weeks old) Syrian hamsters (Mesocricetus auratus) were purchased from Janvier Laboratories (Le Genest-Saint-Isle, France) and kept per two in individually ventilated isolator cages (IsoCage N Bio-containment System, Tecniplast) at 21 °C, 55% humidity and 12:12 day/night cycles. Housing conditions and experimental procedures were approved by the ethics committee of animal experimentation of KU

Leuven (license P065-2020). For infection, hamsters were anesthetized with intraperitoneal injection with ketamine/xylazine/atropine and inoculated intranasally with 50 µL containing 2 x 10^6 $TCID_{50}$ of SARS-CoV-2 WT, strain BetaCov/Belgium/GHB-03021/2020 (EPI_ISL_407976; 2020-02-03) (day 0). Animals were treated either orally with vehicle or vehicle of molnupiravir as negative control, molnupiravir at 200 mg/kg as reference control group or the specified dose regimen of ***iBu*TU** for four consecutive days starting immediately after the intranasal infection. At day 4 post-infection (pi), animals were euthanized for the sampling of the lungs by intraperitoneal injection of 500 µl Dolethal (200 mg/ml sodium pentobarbital, Vétoquinol SA). Lungs were collected for quantification of viral RNA using N2 primers and probes targeting the viral nucleocapsid and infectious virus titers as described before.[35] Infectious virus titers in the lungs were quantified by end-point titration on Vero cells as described before (same reference as RNA quantification). For histological examination, the left lungs were fixed overnight in 4% formaldehyde and embedded in paraffin. Tissue sections (5 mm) were be analyzed after staining with hematoxylin and eosin and scored blindly for lung damage by an expert pathologist. The scored parameters, to which a cumulative score of 1 to 3 was attributed, include the following: congestion, intra-alveolar hemorrhagic, apoptotic bodies in bronchus wall, necrotizing bronchiolitis, perivascular edema, bronchopneumonia, perivascular inflammation, peribronchial inflammation and vasculitis.

[30] Zandi, K.; Amblard, F.; Musall, K.; Downs-Bowen, J.; Kleinbard, R.; Oo, A.; Cao, D.; Liang, B.; Russell, O. O.; McBrayer, T.; et al. Repurposing nucleoside analogs for human Coronaviruses. *Antimicrob Agents Chemother* **2020**, *65* (1).

[31] Tao, S.; Zandi, K.; Bassit, L.; Ong, Y. T.; Verma, K.; Liu, P.; Downs-Bowen, J. A.; McBrayer, T.; LeCher, J. C.; Kohler, J. J.; et al. Comparison of anti-SARS-CoV-2 activity and intracellular metabolism of remdesivir and its parent nucleoside. *Curr Res Pharmacol Drug Discov* **2021**, *2*, 100045.

[32] J.C. Hierholzer, R.A. Killington, 2 - Virus isolation and quantitation, Editor(s): Brian WJ Mahy, Hillar O Kangro, Virology Methods Manual, Academic Press, 1996, Pages 25-46, ISBN 9780124653306

[33] Hurwitz, S. J.; De, R.; LeCher, J. C.; Downs-Bowen, J. A.; Goh, S. L.; Zandi, K.; McBrayer, T.; Amblard, F.; Patel, D.; Kohler, J. J.; et al. Why certain repurposed drugs are unlikely to be effective antivirals to treat SARS-CoV-2 infections. *Viruses* **2024**, *16* (4).

[34] Abdelnabi, R.; Foo, C. S.; Jochmans, D.; Vangeel, L.; De Jonghe, S.; Augustijns, P.; Mols, R.; Weynand, B.; Wattanakul, T.; Hoglund, R. M.; et al. The oral protease inhibitor (PF-07321332) protects Syrian hamsters against infection with SARS-CoV-2 variants of concern. *Nat Commun* **2022**, *13* (1), 719.

[35] Abdelnabi, R.; Boudewijns, R.; Foo, C. S.; Seldeslachts, L.; Sanchez-Felipe, L.; Zhang, X.; Delang, L.; Maes, P.; Kaptein, S. J. F.; Weynand, B.; et al. Comparing infectivity and virulence of emerging SARS-CoV-2 variants in Syrian hamsters. *EBioMedicine* **2021**, *68*, 103403.

[36] Derosa, F.; Heartlein, M. Ribonucleic acids with 4'-thio-modified nucleotides and related methods PCT/US2014/027422

[37] Ahlqvist, G. P.; McGeough, C. P.; Senanayake, C.; Armstrong, J. D.; Yadaw, A.; Roy, S.; Ahmad, S.; Snead, D. R.; Jamison, T. F. Progress toward a large-scale synthesis of molnupiravir (MK-4482, EIDD-2801) from cytidine. *ACS omega*, **2021**, 6(15), 10396–10402.

[38] Johnson, K. A.; Simpson, Z. B.; Blom T. FitSpace explorer: an algorithm to evaluate multidimensional parameter space in fitting kinetic data. *Analytical Biochemistry*, **2009**, 387(1), 30-41

[39] Fagan, S. P.; Mukherjee, P.; Jaremko, W. J.; Nelson-Rigg, R.; Wilson, R. C.; Dangerfield, T. L.; Johnson, K. A.; Lahiri, I.; Pata, J. D. Pyrophosphate release acts as a kinetic checkpoint during

high-fidelity DNA replication by the Staphylococcus aureus replicative polymerase PolC. *Nucleic Acids Res*, **2021**, 49(14), 8324-8338.
